# FOXO1 stimulates tip cell-enriched gene expression in endothelial cells

**DOI:** 10.1016/j.isci.2024.109161

**Published:** 2024-02-16

**Authors:** Yuri Miyamura, Shunsuke Kamei, Misaki Matsuo, Masaya Yamazaki, Shingo Usuki, Keiichiro Yasunaga, Akiyoshi Uemura, Yorifumi Satou, Hiroto Ohguchi, Takashi Minami

**Affiliations:** 1 Divison of Molecular and Vascular Biology, IRDA, Kumamoto University, Kumamoto 860-0811, Japan; 2 Division of Genomics and Transcriptomics, Joint Research Center for Human Retrovirus Infection, Kumamoto University, Kumamoto 860-8556, Japan; 3 Division of Medical Biochemistry, Graduate School of Medical Science, Kumamoto University, Kumamoto 860-8556, Japan; 4 Liaison Laboratory Research Promotion Center, IMEG, Kumamoto University, Kumamoto 860-8556, Japan; 5 Department of Retinal Vascular Biology, Nagoya City University Graduate School of Medical Sciences, Nagoya 467-8601, Japan; 6 Division of Disease Epigenetics, IRDA, Kumamoto University, Kumamoto 860-0811, Japan

**Keywords:** Molecular biology, Cell biology

## Abstract

*Forkhead* box O (FOXO) family proteins are expressed in various cells, and play crucial roles in cellular metabolism, apoptosis, and aging. FOXO1-null mice exhibit embryonic lethality due to impaired endothelial cell (EC) maturation and vascular remodeling. However, FOXO1-mediated genome-wide regulation in ECs remains unclear. Here, we demonstrate that VEGF dynamically regulates FOXO1 cytosol-nucleus translocation. FOXO1 re-localizes to the nucleus via PP2A phosphatase. RNA-seq combined with *FOXO1* overexpression/knockdown in ECs demonstrated that FOXO1 governs the VEGF-responsive tip cell-enriched genes, and further inhibits DLL4-NOTCH signaling. Endogenous FOXO1 ChIP-seq revealed that FOXO1 binds to the EC-unique tip-enriched genes with co-enrichment of EC master regulators, and the condensed chromatin region as a pioneer factor. We identified new promoter/enhancer regions of the VEGF-responsive tip cell genes regulated by FOXO1: *ESM1* and *ANGPT2*. This is the first study to identify cell type-specific FOXO1 functions, including VEGF-mediated tip cell definition in primary cultured ECs.

## Highlights

• Nuclear localized FOXO1 is essential for vessel sprouting and tip cell definition
• FOXO1 and DLL4-NOTCH signaling are mutually inhibited in VEGF-treated ECs
• FOXO1 transactivates tip-enriched gene set with co-enrichment of EC master regulators
• Endothelial FOXO1 associates with EC-specific epigenetic microenvironment

## Graphical abstract

**Figure:**
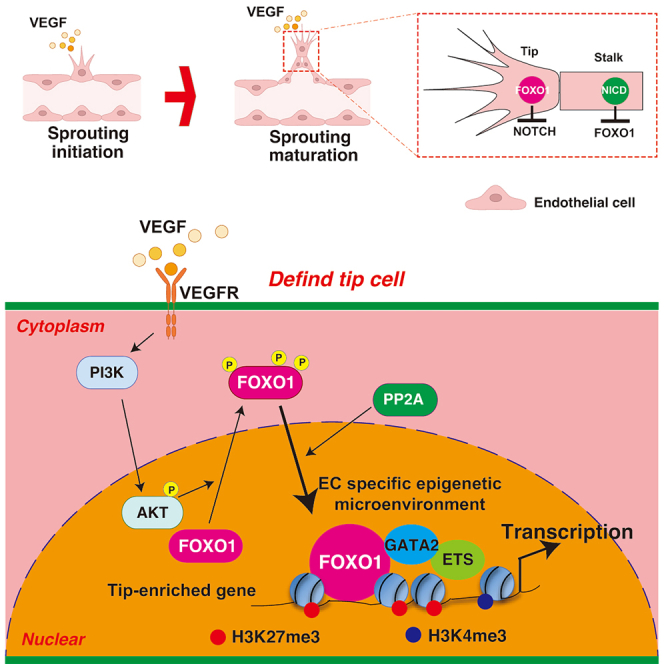

## Introduction

The endothelium is a highly malleable single-cell layer that continuously senses and reacts to the surrounding microenvironmental changes. Vascular endothelial cell growth factor (VEGF) is a cytokine which is expressed in many cell types, including endothelial cell (EC)s-that modulates epigenetically coordinated global gene transcription, subsequently regulating cell permeability, proliferation, and successive angiogenesis.^1^ Angiogenesis starts during EC-migration with a sprouting front (tip) and the following growth-rich (stalk) cell formation.^2^^,^^3^ The tip and stalk cells are dynamically exchanged under three-dimensional culture conditions *in vitro.*^4^ Gene signature associated with tip or stalk cells has been identified; therefore, they are used as markers for the specific cell type. The gene set is well-defined using retina vessel imaging.^2^^,^^5^ VEGF signaling and delta-like (Dll)4-Notch signaling are involved in tip vs. stalk cell separation;^6^ however, the transcriptional system that determines tip or stalk cell specificity in EC is not fully understood.

The *forkhead* box O (FOXO) transcription factor family consists of four members (FOXO1/3/4/6) in mammalian cells. These regulate cellular homeostasis through modulating apoptosis, metabolism, and aging.^7^ However, only the global FOXO1-null mutation leads to embryonic lethality on embryonic day 10.5 through abnormal angiogenesis and placental malformations.^8^^,^^9^^,^^10^ FOXO1 is functionally inactivated by the phosphatidylinositol 3-kinase (PI3K)-AKT pathway via phosphorylation of its serine 256/319 and threonine 24 residues.^11^ In contrast, functional activation and nuclear localization of FOXO1 is regulated by the protein phosphatase, PP2A,^12^ as well as Mst-1/2-,^13^ , and Jun-kinases.^14^ FOXO1 promotes EC quiescence via the antagonistic pathway of MYC signaling under serum-starved or angiogenic-less stabilized vasculature conditions.^15^ Several studies have been investigated the subcellular localization of FOXO1 in response to different extra- and intercellular signals, including VEGF.^16^^,^^17^^,^^18^ Considering the importance of elucidating the mechanism underlying tip vs. stalk definition in the sprouts maturation and FOXO1 activity during VEGF-mediated vessel remodeling, we evaluated the FOXO1-regulated genes using single cell RNA-seq and ChIP-seq analyses in VEGF-treated ECs.

## Results

### Nuclear localized FOXO1 is essential for vessel sprouting and tip cell definition in the VEGF-treated endothelium

**Figure 1:**
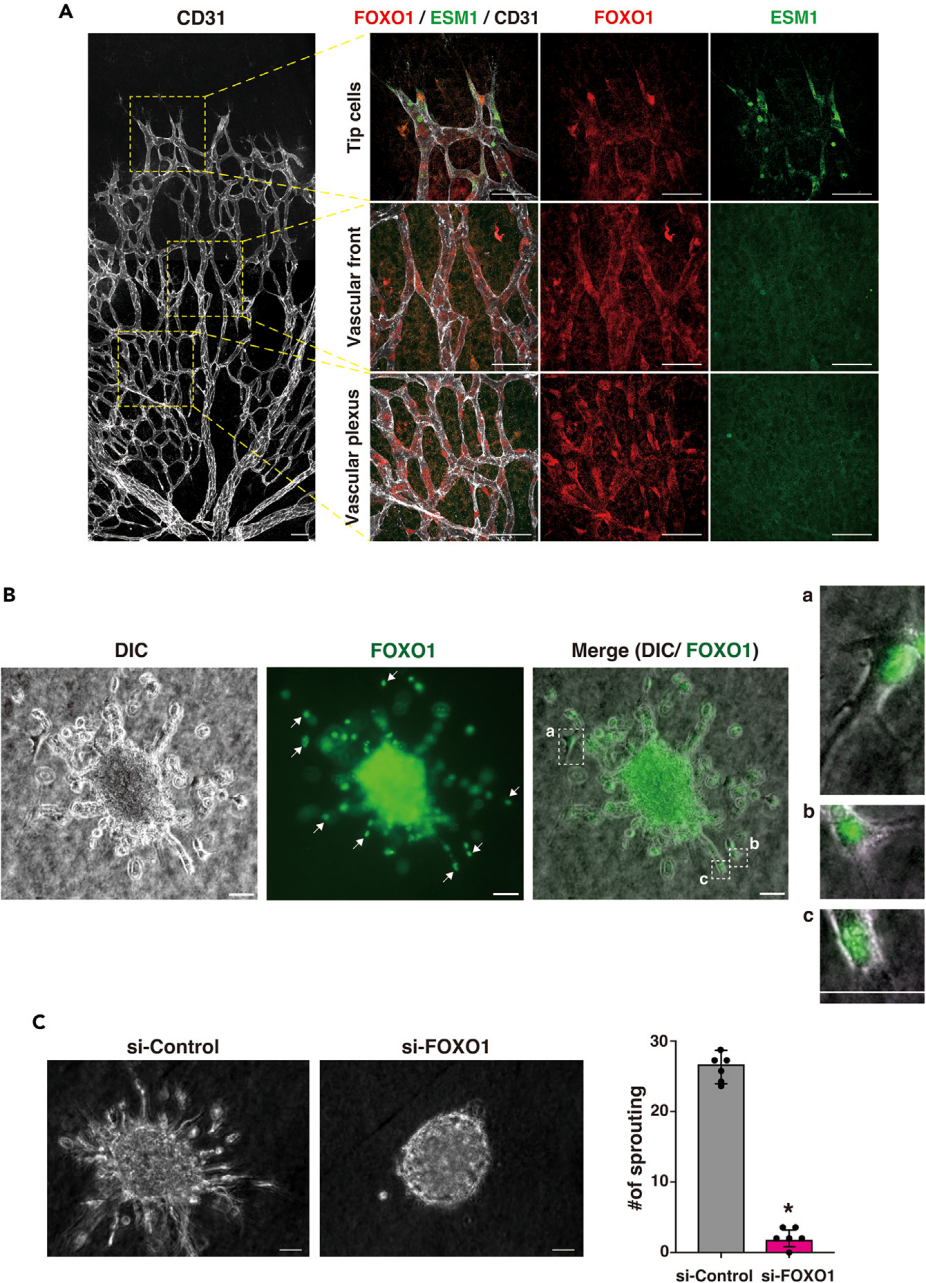
Nuclear-localized FOXO1 is detected in angiogenic fronts which is essential for vessel sprouting (A) Angiogenic proceeding postnatal day-5 mice retina was dissected and immunostained with CD31 for vessel imaging (*left*). Retinas are divided into three areas; tip, vascular front, and vascular plexus, which are enlarged and assessed for the immunostaining using antibodies for FOXO1 (*red*), CD31 (*white*), and ESM1 (*green*). ESM1 is used as the tip marker. Data are representative of three independent experiments. Bar: 50 μm. (B) HUVECs formed spheroids and sprouted in the three-dimensional culture. A bright field differential interference contrast (DIC) image was shown on the *left*. Immunostaining with anti-FOXO1 antibody was shown in *green* color (*middle*). Arrows indicated the cells obtaining nuclear-localized FOXO1. Merged images and the high-power view of spout-front (tip-like) regions, representative in a-c, were shown on the *right*. Bar: 50 μm. (C) HUVECs spheroids were subjected to sprouting assay for 2 days in the presence of siRNA for control or FOXO1. Bar: 50 μm. The *right* graph indicates the number of sprouts from at least 5 independent experiments. Data are shown as mean ± S.D. Black dot represents each individual value. ∗p *<* 0.005 compared with si-Control. See also Figures S1–S3.

FOXO1 is critical for blood vessel formation.^9^ VEGF is a well-known inducer of the initial angiogenesis step, including EC sprouting and tip cell formation.^2^^,^^3^ To verify whether FOXO1 activation correlates with tip cell formation in ECs, postnatal day-5 mouse retina was dissected, and whole-mount immunostaining was performed. Nuclear-localized FOXO1 signals were predominantly enriched at the tip cell region and vascular plexus area, compared with those in the stalk region of cells in the vascular front (Figure 1A), consistent with an earlier report.^19^ The enlarged vascular sprouting front region harbored tip cells with nuclear-localized FOXO1. The FOXO1 signals were co-localized with the tip cell-enriched marker protein, ESM1^20^ (Figure S1A, arrowheads; Figure S1B from the independent specimen). To assess the dynamic changes between the tip- and stalk-like cells,^4^ we performed a spheroid sprouting assay using Human umbilical vein endothelial cells (HUVECs). As shown in Figure 1B, most sprouting fronts obtained FOXO1-positive staining in the nuclear (arrows). As the tip-like cells showed filopodia-like morphologies at the sprouts, protrusion-obtaining cells at the sprouting fronts typically indicated the nuclear-localized FOXO1 signals in the higher magnification (*right* column). Next, to ascertain the functional relevance of FOXO1 in VEGF-stimulated ECs, we evaluated the effect of FOXO1 on budding in HUVECs following FOXO1 knockdown of spheroids in collagen gel. Many cell buds were formed from the EC-spheroids (Figure 1C); these were inhibited by ∼97% in the presence of FOXO1 siRNA (Figures S1C and S1D for the siRNA knockdown effect) compared with that in the si-control.

To evaluate the dynamics of nuclear-cytoplasmic localization of FOXO1, HUVECs were treated with VEGF and analyzed at the indicated time points (Figure S2A). At an earlier time point (30 min) following VEGF treatment, FOXO1 was minimally localized in the nucleus. However, in VEGF treatment for 1, 18 and 24 h later time points, cytosolic FOXO1 significantly re-localized to the nucleus (Figure S2A). In contrast, the nucleus localization ratio of FOXO1 increased marginally following 30 min of TNF-α treatment (Figure S2B), consistent with the previous finding of AKT inhibition.^21^^,^^22^ Pretreatment with the PI3K-AKT inhibitor, LY294002 resulted in a complete loss of the VEGF-triggered extrusion of FOXO1 from the nucleus to the cytosol (Arrowheads in Figure S2C). Phosphorylated FOXO1 is reactivated by various kinases or phosphatases.^23^ In addition to AKT, JNK, MST-1/2, and PP2A are reported to regulate the phosphorylation of FOXO1 in neurons and fibroblasts.^12^^,^^13^^,^^24^ Therefore, to test whether these kinases and phosphatases affect the VEGF-mediated FOXO1 cellular localization in ECs, HUVECs were pre-treated with JNK, MST-1/2, or PP2A inhibitors. The PP2A inhibitor, LB-100, markedly reduced PP2A-mediated FOXO1 re-localization to the nucleus after 1 h; subsets of cells with only cytosolic FOXO1 were observed (shown by arrowheads) (Figure S2D). The cellular localization of FOXO1 was also assessed using immunoblotting. VEGF treatment for 30 min resulted in reduced nuclear FOXO1 and increased cytosol FOXO1 (Figure S3A); this was reversed in HUVECs treated with VEGF for 1 h. Pretreatment with LB-100 retained the FOXO1 in the cytosol; this was accompanied by an increase in phosphorylated FOXO1 both in the nuclear and cytosolic fractions, indicating that PP2A-mediated dephosphorylation process is required for the FOXO1 re-nuclear localization after 1 h VEGF treatment. In contrast, pretreatment with the JNK inhibitor, SP600125 and the Mst1/2 kinase inhibitor, XMU-MP-1, did not interfere with the VEGF-mediated nuclear-cytoplasmic localization of FOXO1 for 1h (Figure S3B). Therefore, VEGF treatment leads to FOXO1 extrusion from the nucleus mainly via AKT signaling; the re-localization of FOXO1 to the nucleus after 1 h is mediated via PP2A activation.

VEGF stimulates DLL4 expression in tip cells, and the subsequent Notch signals are selectively transduced into the stalk cells.^2^ To evaluate the correlation between FOXO1 expression and Notch signaling, HUVECs were transduced with adenovirus harboring the Notch1 intercellular domain (Ad-NICD1). Stalk cell-mimicked NICD overexpression considerably reduced *FOXO1* mRNA expression (Figure S3C). In contrast, *FOXO3* expression was upregulated following Notch signal activation (Figure S3C). Moreover, NICD overexpression resulted in ∼70% reduction of the FOXO1 protein expression in ECs, whereas low dose (2.5 μM) of Notch inhibitor DAPT treatment elevated FOXO1 expression by 1.3-fold (Figure S3D). Much higher dose (25μM) of DAPT oppositely reduced FOXO1 expression, suggesting a biphasic effect of Notch inhibitor in agreement with the previous finding^25^ (Figure S3D). These results indicated that Notch1 suppresses FOXO1 expression in ECs.

### FOXO1 nuclear re-entry after VEGF-mediated extrusion regulates the expression of tip cell-related gene set in the endothelium

**Figure 2:**
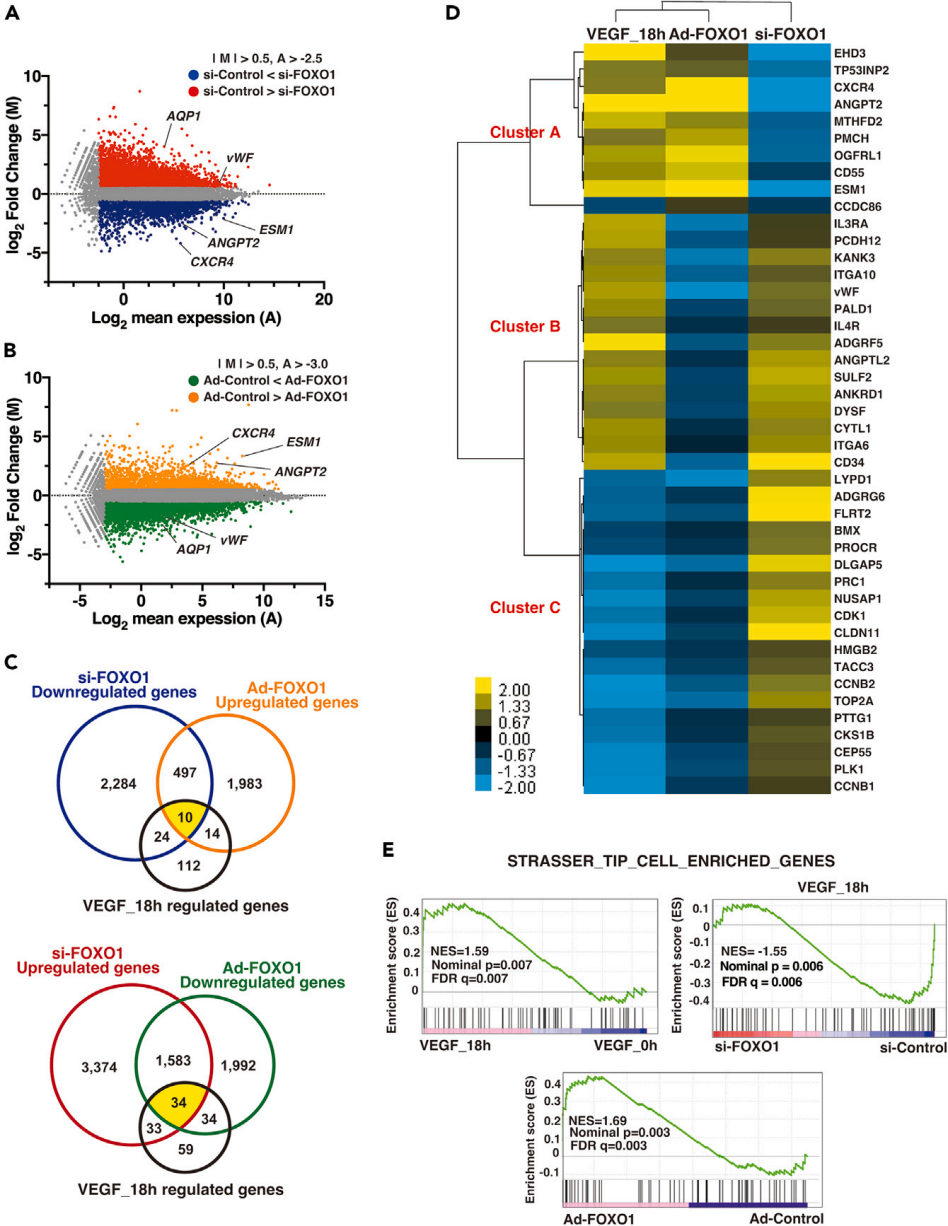
Genome-wide RNA-seq screening of FOXO1 regulated genes in the endothelium (A) MA plot of average gene expression (log_2_, X axis (A)) and the mean of log_2_-fold change (Y axis (M)) of si-FOXO1-mediated up or downregulated genes; these were compared with si-Control HUVECs treated with VEGF for 18 h. *Red* and *blue* dots indicate genes with substantial change in expression with M > 0.5 and A > −2.5. (B) MA plot of average gene expression (log_2_, X axis (A)) and the mean of log_2_-fold change (Y axis (M)) of Ad-FOXO1-mediated up or downregulated genes compared with that in Ad-Control treated HUVECs. *Green* and *orange* dots indicate genes with significantly altered expression with M > 0.5 and A > −3.0. (C) Venn diagram of overlapping si-FOXO1-, or Ad-FOXO1-mediated up or downregulated genes and genes responsive to 18-h VEGF treatment. Each number indicates gene volume. *Yellow* indicates the common responsive gene number. (D) Clustering and heatmap representation of the 44 common FOXO1-regulated genes from *D*. The color intensity indicates the log_2_-fold expression levels: *yellow*-higher and *blue*-lower, relative to the median in *black*. The gene name based on the reference sequence is shown on the *right*. (E) FOXO1-mediated Tip cell enrichment from Gene set enrichment analysis (GSEA). The normalized enrichment score (NES), *P* and *q*-values for the datasets from VEGF 18h vs. 0h (*left*), si-FOXO1 vs. si-control with 18h VEGF treatment (*right*), and Ad-FOXO1 vs. Ad-Control (*bottom*). See also Figure S4 and Tables S1, S2, S3, and S4.

VEGF mediates immediate (within 1 h) gene responses on a genome-wide scale.^26^ NFAT and the downstream early growth response factor transactivate early angiogenic or inflammatory responses in ECs.^27^ In contrast, FOXO1 mainly regulates expression of late response proteins in VEGF-treated ECs.^28^ To validate the regulation of the late response genes by FOXO1 in VEGF-treated ECs, we performed RNA-seq combined with FOXO1 knockdown or overexpression and merged the data with the VEGF-treated HUVEC gene cluster. We first screened si-FOXO1-mediated up and downregulated genes in 18 h VEGF-treated ECs (Tables S1 and S2). Typical tip cell genes, such as *Cxcr4*, *Angpt2*, and *Esm1*, were downregulated, while the stalk cell-enriched genes, *Aqp1* and *vWF*, were upregulated by FOXO1 knockdown (Figure 2A), which is validated by real-time qPCR (Figure S4A). To complement the knockdown experiments, we generated the FOXO1 cDNA harboring T24A, S256A, and S319A mutations to facilitate nuclear translocation and overexpressed it in HUVECs using adenovirus (Figures S4B and S4C). Under this condition, 2,515 and 3,616 genes were up and downregulated in FOXO1-overexpressed ECs, respectively (Table S3). The tip cell-enriched genes, *Cxcr4*, *Esm1*, and *Angpt2*, were the most commonly induced, whereas the stalk genes, *vWF* and *Aqp1*, were inhibited (Figures 2C and S4D). We next selected 160 genes differentially expressed after 18 h of VEGF treatment (Table S4). 27.5% (44/160) of these genes were overlapped with the FOXO1-up or downregulated genes (Figure 2C). Ten genes were categorized as the VEGF-responsive and FOXO1-induced group; 34 genes as VEGF-responsive and FOXO1 repressed group (Figure 2C). Such groups are clustered in Figure 2D. Unbiased clustering revealed that the expression pattern following 18 h of VEGF treatment was more similar to that following the Ad-FOXO1 treatment than to that following the si-FOXO1 treatment. VEGF and FOXO-inducible gene set (cluster A) was predominantly enriched in the EC-specific tip marks (Figure 2E). Interestingly, gene set enrichment analysis (GSEA) demonstrated that VEGF-sustained stimulation led to the marked enrichment of the categorized tip cell-enriched genes,^5^ which are inversely correlated to the FOXO1 knockdown. In contrast, FOXO1 overexpression resulted in the significant enrichment of these tip cell genes in GSEA (Figure 2E). Moreover, Ad-FOXO1 negatively enriched the signatures of the mitotic spindle (Figure S4E), suggesting that FOXO1 promotes the anti-proliferation ways opposing to Notch-mediated stalk signals. Indeed, FOXO1 and Notch activations were mutually inhibited (Figures S3D and S4E). Although FOXO1 expressed various cells, FOXO1 in ECs induced the EC-development-related gene set (Figure S4E). Taken together, these genome-wide data indicate that VEGF-induced FOXO1 activation regulates the EC-specific and tip cell-enriched gene sets.

### VEGF treatment commonly upregulates the tip cell-enriched genes in whole HUVEC subclusters

**Figure 3:**
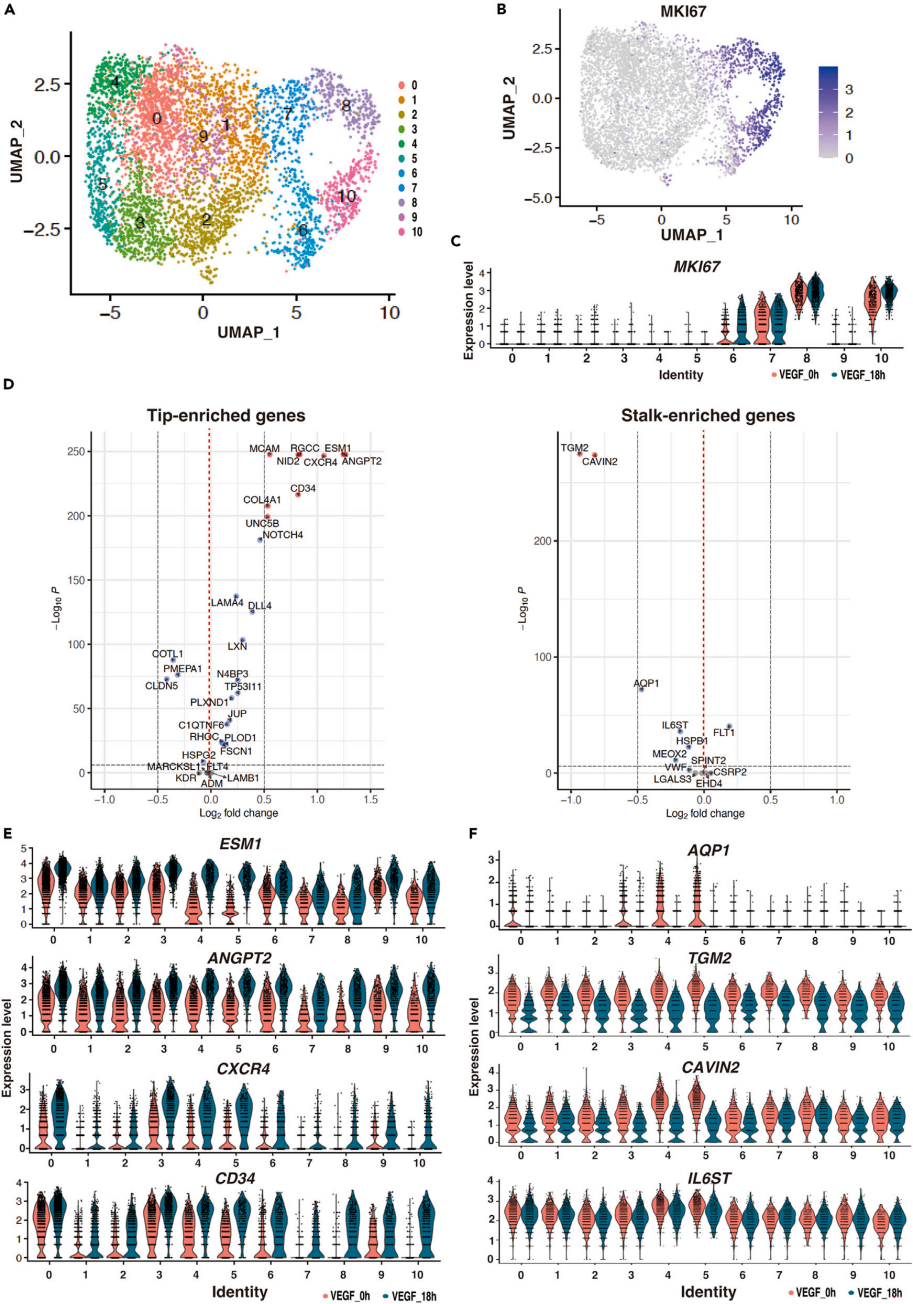
scRNA-seq analysis with tip or stalk cell enriched gene sets in VEGF treated 2D-cultured endothelium (A) UMAP plots colored by ten differential transcriptomic subpopulations. (B) UMAP plots indicate the heterogeneities of HUVECs with VEGF for 18h. Positive (*blue*) and negative (*gray*) of proliferation marks, ki-67. Values are shown in *left* bar relative to *gray*. (C) Violin plots of ki-67 gene expression levels among the subgroups of 1–10. VEGF for 0 h (in *red*) and 18 h (in *green*). (D) Dot plots of pseudo-bulk samples in the scRNA-seq in 0 h vs. 18 h VEGF-treated HUVEC cells. Tip and stalk enriched gene sets were shown in *left* and *right*, respectively. (E and F) Violin plots of expression value for representative tip (*E*) and stalk (*F*) genes in 10 differential subpopulations (red, 0h; green, 18h VEGF treatment). See also Figure S5 and Table S4.

We demonstrated that VEGF treatment induces tip cell-enriched genes via FOXO1 activation in HUVECs. To ascertain whether tip cell-enriched genes are induced only in specific HUVEC subpopulations, we performed single cell (sc) RNA-seq in two dimensional (2D)-cultured HUVECs treated with VEGF using the 10x Genomics platform. By using the Seurat program, we identified ten different subpopulations as plotted in UMAP analysis (Figure 3A). The Ki67-positive proliferative cells were located in clusters 6–8 and 10, whereas ki-67-negative cells were in the rest of the clusters (Figure 3A and 3B). VEGF treatment leads to the upregulation of most of the tip-enriched genes, including CD34 (Figure 3D).^29^^,^^30^ In contrast, most of the stalk-enriched genes were modestly reduced via VEGF (Figure 3D). Among the tip-enriched genes, *ESM1*, *ANGPT2*, and *MCAM* were expressed in most of the clusters in the basal state (Figures 3E and S5A). Meanwhile, *CXCR4*, *CD34*, and typically *C1qTNF6* and *FLT4* were only expressed in the selected clusters, predominantly in the Ki-67 negative group (Figures 3E and S5A). Moreover, VEGF stimulates the expression of the tip-enriched genes in all subpopulations, except for *UNC5b* (Figures 3E and S5A). Among the stalk cell-enriched genes, *AQP1* but not *TGM2*, *CAVIN2*, and *IL6ST*, were detected in some specific populations in basal condition, which were downregulated with VEGF administration (Figure 3F). As shown in a heatmap, three subgroups had unique gene expression patterns both in the presence and absence of VEGF (Figure S5B). Moreover, in the 2D-cultured endothelium, the unique tip mark, DLL4, was expressed uniformly except for subcluster 10, and VEGF induced DLL4 expression in all these subclusters. NOTCH1, 2, and 4 were co-expressed in the DLL4-positive cells (Figures S5A and S5C). These results indicate that tip cell-enriched genes are commonly upregulated in all HUVECs subpopulations after VEGF treatment, providing the rationale for using bulk VEGF-treated HUVECs as tip-like cells.

### FOXO1 preferentially binds at the regulatory regions of the tip cell-enriched genes in the endothelium

**Figure 4:**
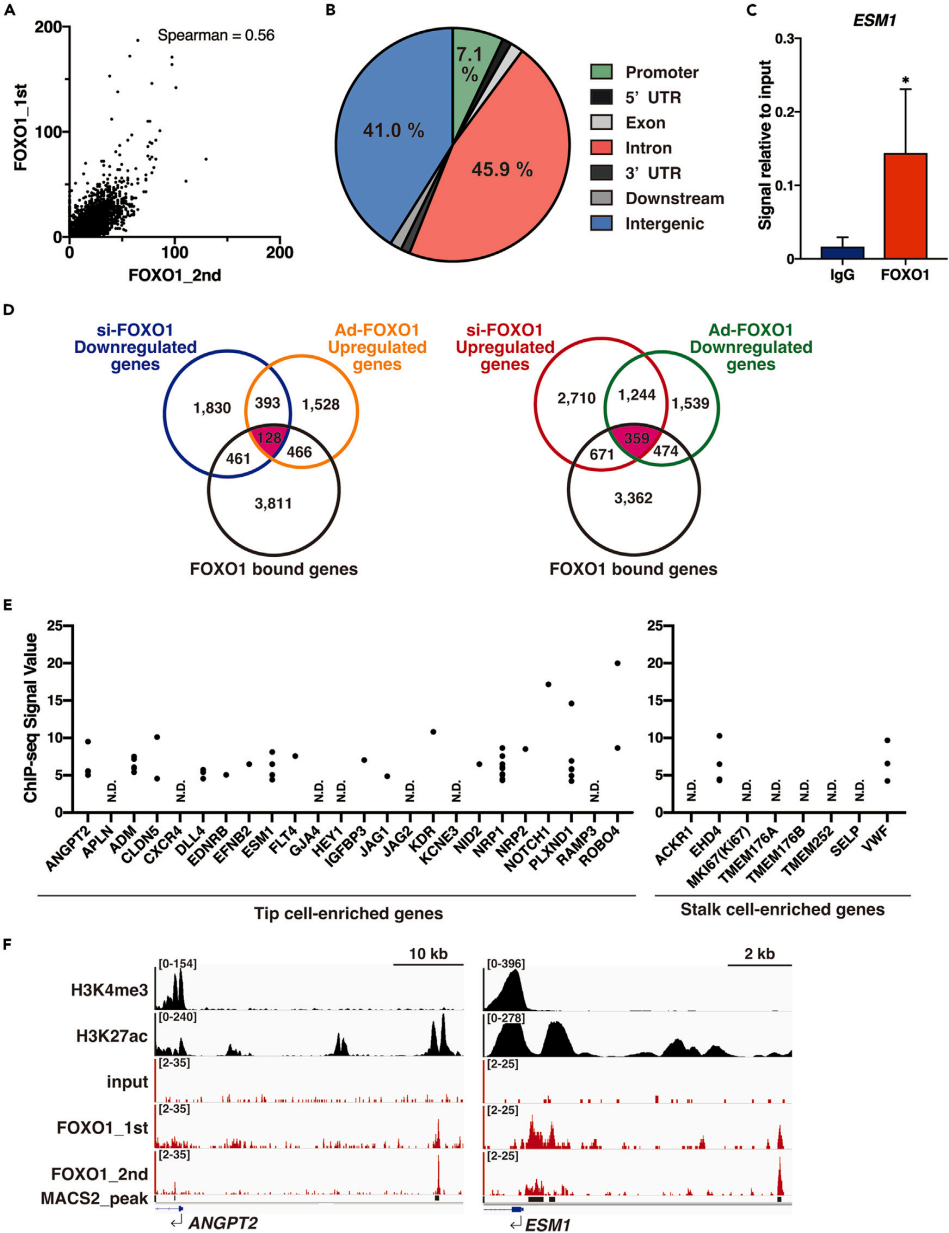
Genome-wide FOXO1-ChIP-seqs analysis in VEGF-treated endothelium (A) Correlation mapping with spearman score of 1^st^ and 2^nd^ FOXO1-ChIP-seqs. (B) Pie-chart of FOXO1-binding genome region in HUVECs using *cis*-regulatory element annotation system. (C) ChIP-qPCR validation of *ESM1* loci from VEGF-treated HUVECs normalized to the input using the FOXO1-specific antibody and the control IgG. (D) Venn diagram with overlapping si-FOXO1- or Ad-FOXO1-mediated up or downregulated genes and the genes involved in the enrichment regions from FOXO1-ChIP-seq. Each number indicates the gene volume. *Purple* indicates the common responsive gene number. (E) FOXO1-ChIP-seq enrichment values are quantified with each tip vs. stalk mark categorized gene loci derived from the ref.^32^ N.D.: not detected. (F) *ANGPT2* and *ESM1* integrated genome view with ChIP-seq enrichment of H3K4me3, H3K27Ac, and two independent FOXO1. Input indicates the negative control for FOXO1-ChIP-seq. MACS_peak bar indicates the significantly enriched region according to the MACS calculation methods. See also Figure S6 and Tables S5, S6, and S7.

RNA-seq following FOXO1 knockdown or overexpression cannot distinguish between direct DNA binding or indirect outcomes. To identify the target genes directly regulated by FOXO1, we performed duplicate ChIP-seqs of endogenously expressed FOXO1 in VEGF-treated HUVECs. MACS_peaks in the whole genome information are summarized in Table S5. The quality of ChIP-seqs was evaluated using the enrichment values from the known FOXO1-binding area at the proximal promoter of HBP1 as the positive control^31^ (Figure S6A) and spearman correlation test (Figure 4A). About two-thirds of FOXO1-enriched signals were localized 2 kb upstream of the 5′-flanking promoter, in the 5′-UTR, and in the intronic region in each gene. The rest were categorized as inter-genes and involved in distal enhancer regions (Figure 4B). FOXO1-ChIP showed more than 10-fold enrichment values for the *ESM1* proximal-promoter region compared with those for the IgG control (Figure 4C). The endogenous FOXO1 ChIP-seq data revealed strong positive signals on the well-known FOXO1 downstream target genes (Figure S6B). The FOXO1-ChIP-seqs were merged with FOXO1 knockdown or overexpressed RNA-seqs (summarized data shown in Table S6); 128 and 359 genes were grouped as FOXO1-bound plus FOXO-upregulated and downregulated genes, respectively (Figure 4D). A functional cluster of each group indicated that FOXO1 can regulate vessel maturation, by modifying cell proliferation and migration capacity and avoiding *anoikis*-mediated cell death (Figure S6C; Table S7). EC-sprouting is initiated by the formation of the tip and stalk cells. To evaluate whether FOXO1 directly binds to the regulatory regions of a tip cell-enriched gene, ChIP-seq data were clustered with a defined tip vs. stalk cell-enriched gene set. VEGF-activated FOXO1 preferentially bound to the tip cell-enriched genes group. In addition, FOXO1 bound a few stalk cell-enriched genes; this association resulted in downregulation of these gene expression (Figures 4E and S7A). Integration genome viewer showed that FOXO1 binding was co-localized with the H3K4me3-positive proximal-promoter and with the H3K27ac-enriched enhancer region of EC-specific and typical tip cell-enriched genes, such as *ESM1*, *ANGPT2, ROBO4, and CD34* (Figures 4F and S6D). In addition, stalk cell-gene loci, such as *AQP1*, *vWF*, and *EHD4*, were enriched with FOXO1 signals surrounded by open chromatin marks, such as H3K4me3 and H3K27ac (Figure S6E).

**Figure 5:**
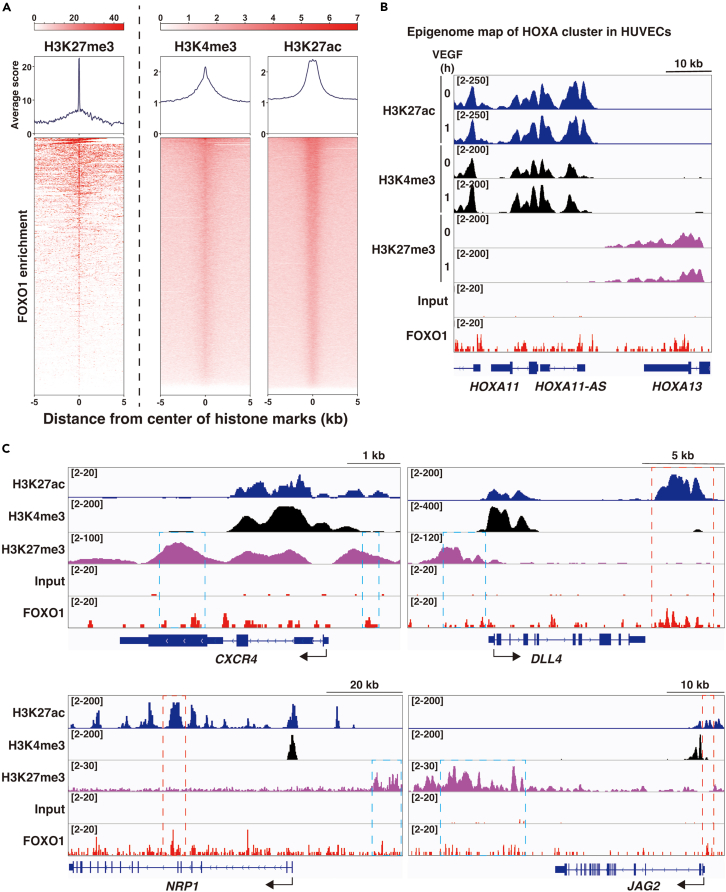
FOXO1 binds to both closed and open chromatin regions as a pioneer factor in the VEGF treated endothelium (A) FOXO1 enrichment patterns are compared with those of H3K27me3, H3K4me3, and H3K27ac histone codes. The FOXO1 enrichment around the peaks of each histone code (±5 kb) in HUVECs. (B) Integrated genome view of HOXA11 with 13 genome regions. Histone code information of H3K27ac (*blue*), H3K4me3 (*black*), and H3K27me3 (*purple*) from cells treated with VEGF for 0 h and 1 h, with adjusted peak levels shown at the *upper left*. Input indicates the negative control for FOXO1-ChIP-seq. (C) Integrated genome view of the indicated tip cell enriched gene loci. *Blue* and *red* broken rectangles indicate FOXO1 bound at the H3K27me3 and H3K27ac enriched areas, respectively. FOXO1 enrichment on the *CXCR4* genome locus was derived from single ChIP-seq data because of the failure to get profound FOXO1 enrichment on the locus, using MACS peak call with another ChIP-seq. See also Figure S7.

RNA-seq results indicated that FOXO1 binding results in both up and downregulation of the target genes. ChIP-seq with FOXO1 showed a narrow solid peak for some targets, such as *ESM1* and *ANGPT2* gene loci; in addition, it showed varying peak patterns for several other targets. Therefore, to evaluate the correlation with the chromatin environment, FOXO1 binding patterns were merged with the histone code information. FOXO contains the *forkhead* domain, which can displace the linker histone H1, enabling access to the enhancer nucleosome in chromatin as a pioneer factor.^33^ The FOXO1 enrichment scores were much higher in polycomb complex (PRC)2-mediated H3K27me3 than in the open chromatin marks, H3K4me3 and H3K27ac; however, all the enrichment scores were significant (Figure 5A). The HOXA cluster in HUVECs were clearly divided by the open or closed chromatin. *HOXA11* and its antisense gene are open, accompanied by H3K27ac and H3K4me3, whereas HOXA13 is closed, accompanied by H3K27me3. FOXO1-ChIP-seq signals were observed equally even in the PRC2-mediated closed chromatin (Figure 5B). We then analyzed the functionally essential genes in EC with FOXO1 mild associations. Among the tip and stalk cell gene set, co-enrichment of H3K27me3 and FOXO1 was noted in a subset of tip cell-enriched genes, including *CXCR4* (shown with a *blue* rectangle). Co-enrichment of conventional H3K27ac-enhancer mark and FOXO1 was observed in other tip cell genes (shown with a *red* rectangle) (Figure 5C). Co-enrichment of H3K27me3 and FOXO1 was not found in the stalk cell genes (data not shown). In addition, FOXO1 did not interact with H2AK119Ub and EC-unique bivalent region, as demonstrated in the gene loci of *EGR2* and H3K9me3-enriched gene adjacent to the centromere region, *NR110759* (Figure S7B). Genome-wide screening of endogenous FOXO1 via ChIP-seq and histone-code profiling suggested that FOXO1 is a pioneer factor similar to other members of the FOX family and that it selectively associates with the tip cell-enriched genes by altering the chromatin microenvironment. Dynamic histone marking changes were not observed in the presence or absence of VEGF treatment in the case of FOXO1-associated gene set. However, FOXO1-mediated chromatin microenvironmental changes could lead to the association of other positive or negative transcription factor complexes, which might up or downregulate the target genes, respectively, as observed in the RNA-seqs analysis.

### Endothelial FOXO1 associates with EC-specific epigenetic microenvironment

**Figure 6:**
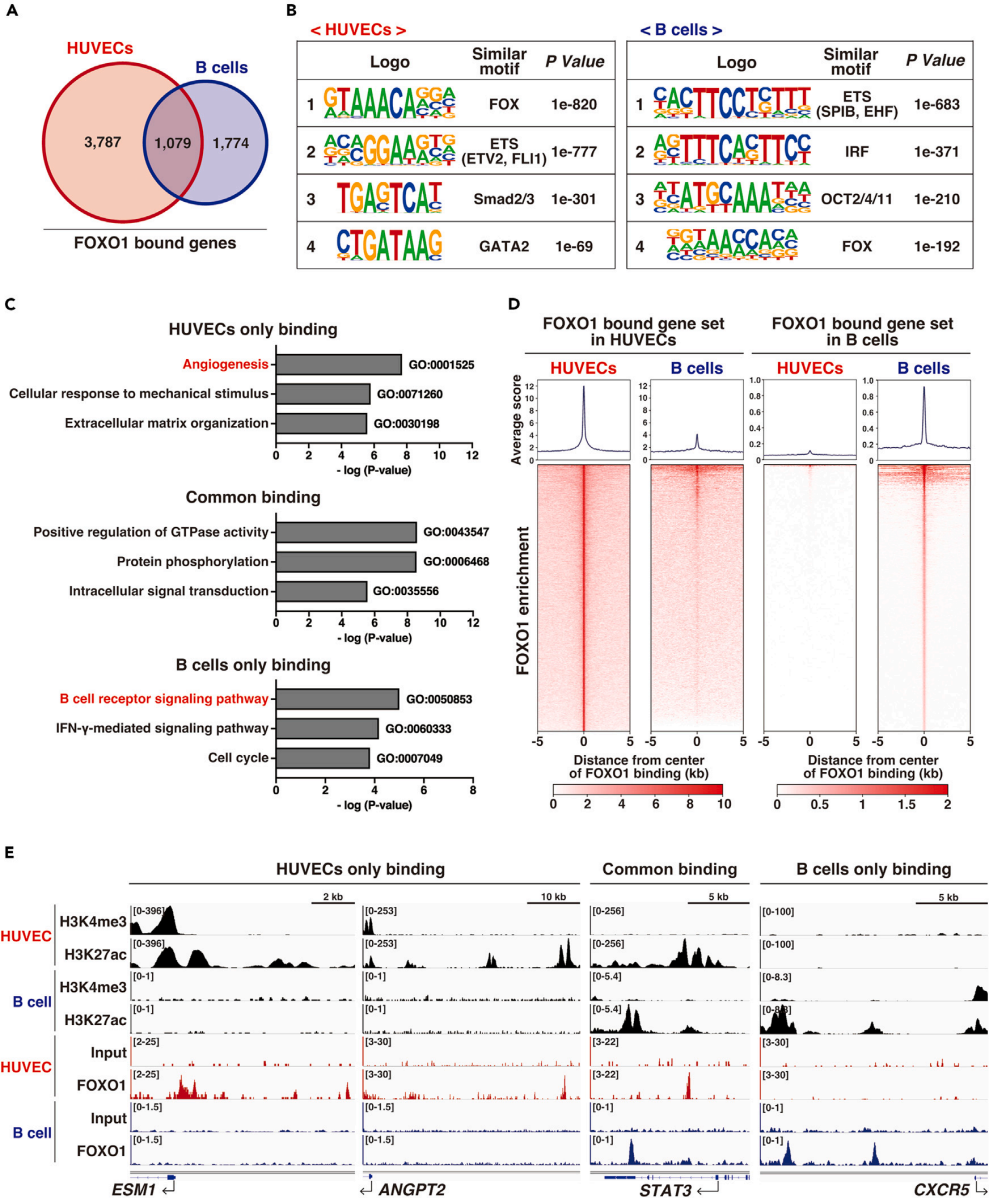
FOXO1 expressed in ECs preferentially bind to EC-selective expressed genes (A) Venn diagram depicting the overlap of FOXO1 binding genes between HUVECs and B cells. (B) MEME co-enrichment analysis of FOXO1 ChIP-seq results from HUVECs (*left*) and B cells (*right*). The position-weight matrix indicates enriched sequences. The *p* value indicates the probability that the *de novo* enriched sequences obtained from ChIP-seq matched to the known consensus motifs by chance. (C) HUVEC- or B cell-specific and common enriched gene sets are subjected to Gene Ontology (GO) analysis. In each case, up to three GO terms with the highest significance and the corresponding *p* values are shown in the graph. (D) FOXO1 enrichment patterns and the scores in HUVECs (*left*) and B cells (*right*) around FOXO1-bound regions (±5 kb) in each cell type are shown. Heatmap density around the same genome loci is shown in the *lower* panel. (E) Integrated genome view of the representative HUVEC- or B-cell specific and common FOXO1 binding patterns. H3K4me3 and H3K27ac ChIP-seqs are shown as the proximal active promoter and enhancer mark, respectively. Input indicates the negative control for FOXO1-ChIP-seq from each cell. See Also Figure S8 and Tables S8 and S9.

The genome-wide screening using FOXO1-RNA-seqs and -ChIP-seqs revealed that FOXO1 is a critical regulator of EC-specific tip cell enriched genes. To test whether such FOXO1 binding pattern is unique to ECs under the cell-specific chromatin microenvironment, we compared them with the results from the parallel ChIP-seq of B cells.^34^ FOXO1 binding patterns were not similar, even though FOXO1 was expressed in both cells (Figure 6A). The co-enrichment MEME analysis suggested that the FOXO1 in ECs interacted specifically with EC-defined ETS and GATA transcription factors at the FOXO1-bound genome region. In contrast, FOXO1 in B cells interacted with lymphocyte-specific ETS and IRF on the genome (Figure 6B). Functional GO clustering revealed that pro- and anti-angiogenesis-related genes are selectively bound by FOXO1 in ECs, while the B cell-specific signal was unique to FOXO1 ChIP in B cells. The binding of FOXO1 to genes related to ubiquitously necessary signals was detected in both cells (Figure 6C; Table S8). This FOXO1-ChIP-seq pattern indicates that binding of FOXO1 could be cell type-specific (Figure 6D; Table S9). EC-specific FOXO1 binding regions (EC-specific tip cell gene; *ESM1* and *ANGPT2*, and EC-specific expressed marker; *CDH5* and *CLDN5*) were enriched with H3K4me3 and H3K27ac at the proximal-promoter and enhancer regions in HUVECs, but not in B cells (Figures 6E and S8 *left*). In contrast, B cell-specific functional gene loci (*CXCR5*, *BLK*, and *CD38*) had unique FOXO1 binding and histone modification patterns specifically in B cells (Figures 6E and S8 *right*). The universally valid FOXO1 target gene loci (*STAT3* and *JAK1*) exhibited common FOXO1 binding even at different enhancer regions marked by H3K27ac (Figures 6E and S8 *middle*). Therefore, FOXO1 binds to different genome regions in a cell type-specific manner modulated by the existing epigenetic microenvironment, though FOXO1 is expressed ubiquitously.

### FOXO1 binding is essential for EC-specific gene transactivation

**Figure 7:**
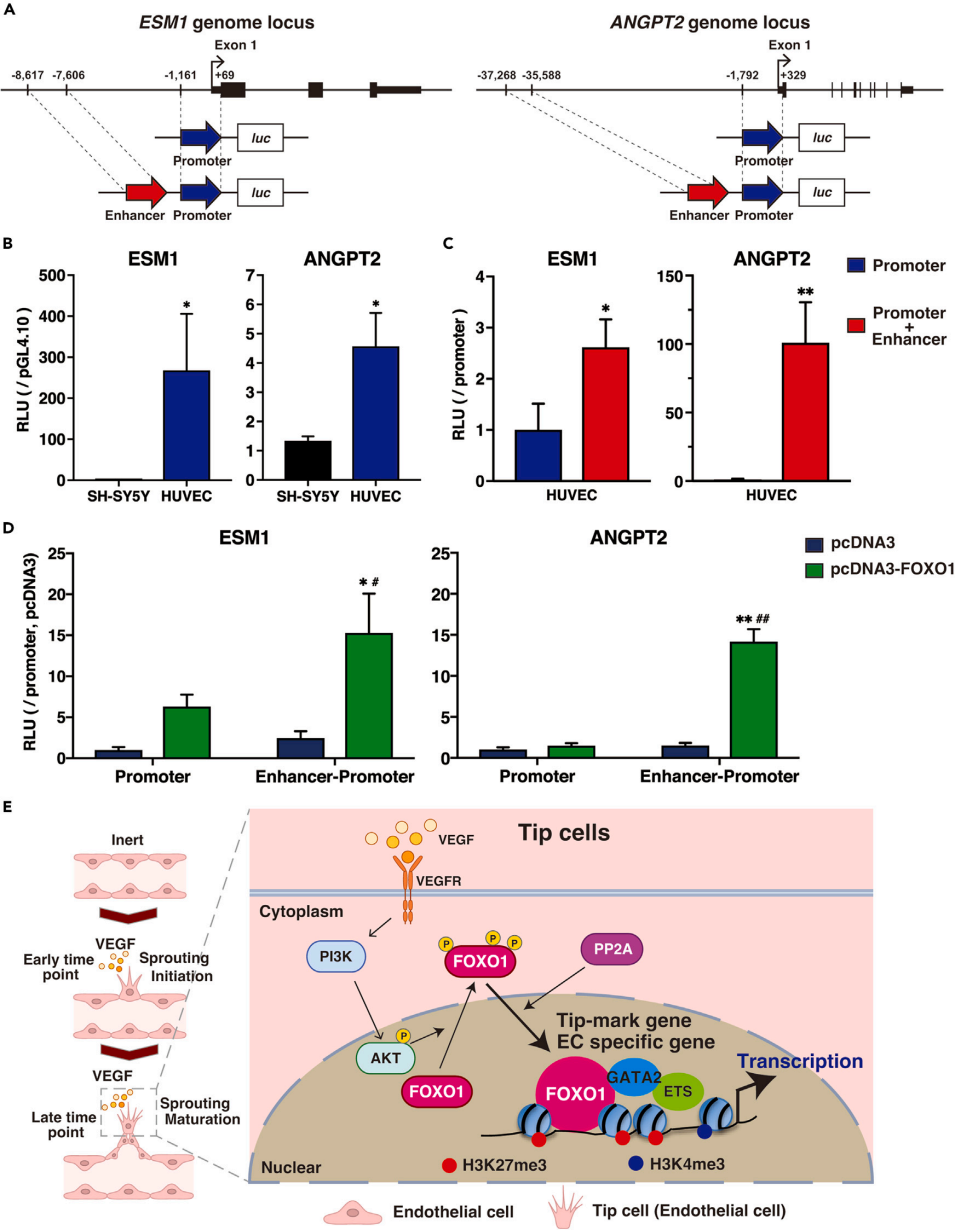
FOXO1-bound enhancers played cell-type specific promoter activation in the endothelium (A) Schematic representation of *ESM1* and *ANGPT2* genome locus and the construction for luciferase reporter analysis with each FOXO1-bindable promoter and promoter+H3K27ac-positive enhancer. (B) ESM1 and ANGPT2 promoter activities in comparison with that of promoter less pGL4.10 in neuronal SH-SY5Y cells or HUVECs. The results show the mean ± S.D. of luciferase right units (RLU) obtained in triplicate from three independent experiments. ∗p < 0.01 compared with the activity from SH-SY5Y. (C) ESM1 and ANGPT2 enhancer activities relative to each promoter in HUVECs. The results show the mean ± S.D. of RLU obtained in triplicate from three independent experiments. ∗p < 0.01 and ∗∗p < 0.005 compared with each promoter alone. (D) Luciferase reporter activity of ESM1 or ANGPT2 promoter alone and promoter + enhancer. COS7 cells were co-transfected with *luc*-reporter plasmids and plasmids that constitutively express nuclear-localized FOXO1. The data indicate the mean ± S.D. of RLU relative to that in control obtained in triplicate from three independent experiments. ∗p < 0.05 and ∗∗p < 0.0005 compared with each mock vector control. #p < 0.01 and ##p < 0.0005 compared with FOXO1 overexpressed promoter alone. (E) Schematic illustration of VEGF-mediated sprouting in ECs. PI3K-mediated nuclear re-localization of cytosol-exported FOXO1 via PP2A, which transactivates tip cell-enriched and EC-specific genes through chromatin remodeling. See also Figures S9 and S10.

The expression of VEGF-inducible tip cell enriched genes (*ESM1* and *ANGPT2*) explicitly depends on EC-specific FOXO1 binding. This is the first study to identify FOXO1 bindable distal enhancers using endogenous FOXO1 ChIP-seqs. Luciferase reporter assay was used to evaluate whether the direct binding of FOXO1 to *ESM1* or *ANGPT2* led to the transcriptional activation of these genes. The promoter and enhancer regions of each gene were connected, and *de novo* enhancer-promoter constructs were generated (Figure 7A). The promoter regions of *ESM1* and *ANGPT2* were functional in the ECs, but not in the neuronal SH-SY5Y cells (Figure 7B). The promotor activity was enhanced by the addition of the enhancer region (Figure 7C). To examine whether FOXO1 mediates these activities, we overexpressed FOXO1 and assessed the promotor and enhancer activities. Overexpressed FOXO1 transactivated *ESM1* promoter activity, which was further enhanced in the presence of the enhancer region (Figure 7D *left*). Similarly, FOXO1 overexpression resulted in enhanced *ANGPT2* promoter activation when the defined enhancer region was ligated (Figure 7D *right*). We next sought to validate the enhancer activity of the *ANGPT2* using CRISPR-Cas 9 system. Because of technical difficulty, the efficacy of genome editing was very low in HUVECs, but we confirmed partial deletion of the enhancer region by Sanger sequencing (Figures S9A–S9D). As expected, lentiviral CRISPR-mediated-deletion of FOXO1-binding *ANGPT2* enhancer region modestly but significantly decreased *ANGPT2* mRNA expression in VEGF-treated HUVECs (Figure S9E). Taken together, these data indicated that EC-specific FOXO1 association with the genome is required to upregulate the functionally essential genes for the VEGF-mediated tip/stalk definition and the subsequent maturation.

## Discussion

EC sprouting is promoted with tip and the following stalk cell definition. Tip and stalk cells are variable, and expression of each specific marker gene is modulated mainly by the epigenetic regulation under the EC maturation process. VEGF is a well-known EC sprout guidance factor that forms tip cells in the sprout front. Tip cells are not proliferative, but stalk cells proliferate well by the NOTCH signal activation.^3^ In this study, we evaluated the dynamics and function of endogenous FOXO1 and revealed its dynamic nuclear-cytoplasmic translocation in the presence of VEGF; in addition, we uncovered FOXO1-mediated gene regulations on a genome-wide scale. GSEA indicated that VEGF induces the tip cell gene signature through the FOXO1 activation. FOXO1-activated ECs inhibited both cell proliferation and NOTCH signals, consistent with the tip cell function.

It has been well-considered that DLL4 in tip cells and the subsequent Notch signals are selectively transduced into the stalk cells.^2^ Beyond the membrane-bound Notch ligand-mediated lateral inhibition theory, it has also indicated that Dll4 was contained in exosomes that induce capillary sprout retraction in the 3D microenvironment.^35^ Moreover, in the 2D-cultured endothelium of our scRNA-seq, DLL4 and NOTCH genes were co-expressed in the same cells (Figure S5). These data suggest that once the cell is fated to be a tip cell, DLL4 in the cell would be better to downregulation to block the Notch signal activation. Interestingly, we have shown that VEGF starts the DLL4 upregulation within 1 h. But at the later time of VEGF treatment, re-nuclear localized FOXO1 inhibited the DLL4 upregulation (Tables S3 and S4). Consistent with scRNA-seq in HUVECs, the epigenome map in the *DLL4* locus revealed both H3K4me3 and H3K27me3 enrichments in a different area, which suggests that the existence of a mixture of DLL4-positive and -negative cells but not the bivalency (Figure S10). Our ChIP-seq data indicate the FOXO1 significantly enriched at the 3′-region of *DLL4* where the co-enrichment of EC-defined master regulators, FLI1 and ERG, is observed in our ChIP-seq.^36^ Further experiments would be needed, but it is possible that VEGF-mediated FOXO1 could be associated with the *DLL4* locus and modify its expression via the interaction of transcription factors (Figure S10).

FOXO is a subfamily member of the Fox group, which contains a winged-helix DNA-binding domain that mimics the DNA-binding region of the linker H1 histone. It could bind directly to the condensed ‘closed’ chromatin and is, therefore, known as a pioneer factor.^33^^,^^37^ FoxA, a unique pioneer factor, enables rearrangement of the chromatin to keep the enhancer nucleosome accessible, allowing the binding of other tissue-specific transcription factors.^38^^,^^39^ Our study suggested that FOXO1 might function as a pioneer factor in the endothelium although further studies are required for providing this notion.

FOXO1 preferentially binds to the regulatory regions of the tip cell and EC-specific functional genes. Motif analysis suggested that FOXO1 in EC is co-enriched with EC pioneer and master regulator transcription factors, such as Etv2, Fli1, and Gata2, leading to EC-specific angiogenesis or VEGF-signaling.^36^^,^^40^^,^^41^ However, FOXO1 in B cells was associated with the B cell pioneer, Spi-B,^42^ mediating the B cell-specific FOXO1 function. Therefore, FOXO1 is required not only for the cell development process, but also for the functioning of fully differentiated ECs, which is exemplified by the regulation of VEGF-related genes in association with epigenetic modulation.

VEGF triggers transient nuclear export of FOXO1 via the PI3K-AKT phosphorylation axis; it then stimulates FOXO1 re-localization to the nucleus via phosphatase PP2A at later time points. Kim. et al. also showed that the MST1-FOXO1 cascade induces tip cell formation and promotes angiogenesis under hypoxic conditions.^19^ In our study, MST1/2 inhibitor did not greatly affect FOXO1 re-entry to the nucleus. This difference might be derived from the experimental conditions since our study was performed under normoxic condition where MST1 might not be activated. We found such the activated FOXO1 preferentially binds to the regulatory regions of the tip cell enriched and EC-specific functional genes (schematically shown in Figure 7E). The genome-wide analysis suggests that the lethality of FOXO1 null mutants, owing to vascular remodeling error, could at least be partially attributed to the defect in tip-mark definition and in the subsequent vessel sprouting associated with FOXO1-mediated transcriptional regulation.

### Limitations of the study

We elucidated the mechanisms of FOXO1-mediated gene regulation using comprehensive mapping of transcriptomes and histone modifications in fully differentiated ECs. However, whether FOXO1 pioneering is universally applicable to the pathogenesis in various ECs under the vascular bed in different organs remains unknown. Our proposed model shows that persistent VEGF signaling-stimulated tip cell formation and sprouted EC maturation predominantly occurred via re-localized nuclear FOXO1. This is in sharp contrast with our recent report of NFAT-mediated immediate-early VEGF signaling and its related epigenetics.^26^ Nonetheless, the dynamics of epigenome modifiers and transcription factors, including FOXO1, must be examined further to understand sustained VEGF signaling in ECs. Thus, dynamic genome-wide analysis involving metabolomics, proteomics, and scRNA-seq analysis comparing tips and stalks could accurately address VEGF-mediated persistent signaling and the subsequent EC sprouting.

## STAR★Methods

### Key resources table

**Table:** 

REAGENT or RESOURCE	SOURCE	IDENTIFIER
**Antibodies**		

FOXO1	Cell signaling technology	Cat# 2880; RRID: AB_2106495
FITC-conjugated CD31	BD Pharmingen	Cat# 555445; RRID: AB_395838
CD31	BD Pharmingen	Cat#550274; RRID: AB_393571
ESM-1	R&D systems	Cat# AF1999; RRID: AB_2101810
FOXO1	Abcam	Cat#ab39670; RRID: AB_732421
Ser256 phosphorylated FOXO	Cell Signaling Technology	Cat#9461; RRID: AB_329831
β-actin	Sigma-Aldrich	Cat# A1978; RRID: AB_476692
Lamin A	Santa Cruz Biotechnology	Cat#sc-20680; RRID: AB_648168
β-tubulin	WAKO	Cat#014-25041; RRID: AB_2650453
HRP-conjugated anti-rabbit IgG	Sigma-Aldrich	Cat#A9169; RRID: AB_258434
HRP-conjugated anti-mouse IgG	Sigma-Aldrich	Cat#A9044; RRID: AB_258431
Phalloidin iFluor™647 Conjugate	Cayman	Cat#20555, RRID: AB_2620155
Alexa Fluor™488-conjugated anti-FITC	Invitrogen	Cat#A11096; RRID: AB_221588
Goat anti-Rabbit IgG, Alexa Fluor™594	Invitrogen	Cat#A11037; RRID: AB_2534095
Donkey anti-Rat IgG, Alexa Fluor™488	Invitrogen	Cat#A21208; RRID: AB_2535794
Donkey anti-Goat IgG, Alexa Fluor™647	Invitrogen	Cat#A21447; RRID: AB_10925072

**Bacterial and virus strains**		

T24A/S256A/S319A FOXO1-IRES-EGFP adenovirus	This paper	N/A
EGFP adenovirus	This paper	N/A
*ANGPT2* enhancer region-targeting CRISPR-Cas9 & gRNAs #1 (#1-5’; 5’-ACCTCTGAC TGAGGCACGTT-3’, #1-3’; 5’-TGAAGTGT TAGGGCGCCTTT-3’) lentivirus	VectorBuilder	VB230331
*ANGPT2* enhancer region-targeting CRISPR-Cas9 & gRNAs #2 (#2-5’; 5’-AGCTGGAG ATGTGCCGCCAA-3’, #2-3’; 5’-CCGGCG GGCGGCTTCACGAC -3’) lentivirus	VectorBuilder	VB230331
EGFP control lentivirus	VectorBuilder	VB010000

**Chemicals, peptides, and recombinant proteins**		

EGM™-2 bullet kit	LONZA	CC-3162
Dulbecco's modified Eagle's medium (DMEM)	WAKO	041-30081
FBS	Sigma-Aldrich	172012
Opti-MEM® I Reduced Serum Medium	gibco	31985-062
Hank’s buffered saline (HBSS)	Nacalai	17459-55
Human VEGF-A	WAKO	229-01313
Human TNF-α	Peprotech	300-01A
LB100	Selleck	S7537
LY294002	Calbiochem	440202
SP600125	WAKO	197-16591
XMU-MP-1	MedChemExpress	HY-100526
Everolimus	WAKO	058-00456
RNAi MAX	Invitrogen	13778075
Sepasol-RNA I Super G	Nacalai	09379-55
THUNDERBIRD® SYBR® qPCR Mix	TOYOBO	QPS-101
PrimeScript RT Master Mix	Takara	RR036A
Type I-A collagen gel	Nitta-gelatin	638-00781
Mitomycin C	Kyowa Kirin	057039107
Formaldehyde	Nacalai	09154-85
FuGENE HD	Promega	E2311
Lipofectamine 2000	Invitrogen	11668019
Accumax	Innovative Cell Technologies	AM105
PrimeSTAR Max	Takara	R045A
DAPT	WAKO	043-33581
Protein Blocker	Agilent, DAKO	X090930

**Critical commercial assays**		

Adeno-X Rapid Titer Kit	Takara	632250
ISOSPIN Cell & Tissue RNA Kit	Nippon gene	314-08211
TruSeq Stranded mRNA Library Prep Kit	Illumina	20020594
Simple ChIP Enzymatic Chromatin IP Kit	Cell Signaling Technology	9003
NEB Next Ultra II DNA Library Prep Kit for Illumina	New England Biolabs	E7645
GenNext NGS Library Quantification kit	TOYOBO	NLQ-101
Chromium NextGEM Single Cell 3' Kit v3.1	10X Genomics	PN-1000269

**Deposited data**		

The reference Series of all raw data	This paper	GEO: GSE220509
RNA-seq raw data of CA-FOXO1	This paper	GEO: GSE220503
RNA-seq raw data of si-FOXO1	This paper	GEO: GSE220504
RNA-seq raw data of VEGF treatment	This paper	GEO: GSE220505
ChIP-seq raw data of FOXO1	This paper	GEO: GSE220508
scRNA-seq raw data of VEGF treatment	This paper	GEO: GSE235885
ChIP-seq data for FOXO1 in germinal center B cells	Dominguez-Sola D et al. (2015)^34^	GSM1668935
ChIP-seq data for H3K4me3 in HUVECs	Wang S et al. (2019)^43^	GSM2947421
ChIP-seq data for H3K27ac in HUVECs	Wang S et al. (2019)^43^	GSM2947429
ChIP-seq data for H3K27me3 in HUVECs	Wang S et al. (2019)^43^	GSM2947425
ChIP-seq data for VEGF-treated H3K4me3, H3K9me3, H3K27me3, H3K27ac, and H2AK119Ub	Kanki Y et al. (2022)^26^	GEO: GSE159075
ChIP-seq data for H3K4me3 in B cells	Pasqualucci L et al. (2015)	GSM1648034
ChIP-seq data for H3K27ac in B cells	Pasqualucci L et al. (2017)	GSM2386720

**Experimental models: Cell lines**		

HUVEC	LONZA	C2519A
Cos-7	ATCC	CRL-1651
SH-SY5Y	ATCC	CRL-2266

**Oligonucleotides**		

FOXO1 siRNA #1 (si-FOXO1 #1) 5’-GAAUUCAAUUCGUCAUAAU-3’	Sigma-Aldrich	SASI_Hs01_00076732
FOXO1 siRNA #2 (si-FOXO1 #2) 5’-GUAUAACUGUGCGCCUGGA-3’	Sigma-Aldrich	SASI_Hs01_00076733
Validation for CRISPR deletion of ANGPT2 enhancer region, No.1 FW 5’- CGGTCGTGAGGAATGTCGTT -3’ RV 5’- AGCGATTCTGAACGGTGCAT -3’	This paper	N/A
Validation for CRISPR deletion of ANGPT2 enhancer region, No.2 FW 5’- CACCAGAGACTCCGTGTACC -3’ RV 5’- CACTCACCCTTGATGGGAGC -3’	This paper	N/A
qPCR primers	See Table S10	N/A
ChIP primers	See Table S10	N/A

**Recombinant DNA**		

T24A/S256A/S319A FOXO1-Gateway pENTR	This paper	N/A
pA/CMV/V5-DEST Gateway Vectors	Invitrogen	V49320
pIRES2-EGFP	Clontech	6029-1
Gateway pENTR 4 Dual Selection Vector system	Invitrogen	A10465
Human *ESM1* promoter-pGL4.10	This paper	N/A
Human *ANGPT2* promoter-pGL4.10	This paper	N/A
pGL4.10	Promega	E6651

**Software and algorithms**		

featureCounts	Liao Y et al. (2014)	https://sourceforge.net/projects/subread/files/subread-1.5.2/
EBSeq (Bioconductor)	Leng N and Kendziorski C (2022)	https://bioconductor.org/packages/release/bioc/html/EBSeq.html
Gene Cluster 3.0	Stanford University	http://bonsai.hgc.jp/∼mdehoon/software/cluster/
Java Tree View version 1.2.0	Stanford University	https://java-treeview.soft112.com/
GSEA	UC San Diego and Broad Institute	https://www.gsea-msigdb.org/gsea/index.jsp
MACS2 (Model-Based Analysis of ChIP-seq)	Zhang Y et al. (2008)	https://github.com/macs3-project/MACS
HOMER	Heinz S et al. (2010)	http://homer.ucsd.edu/homer/
ChIP-Atlas	Oki S et al. (2015)	https://chip-atlas.org/
Documents for computational processing (ChIP-Atlas)	Oki S et al. (2018)^44^	https://github.com/inutano/chip-atlas/wiki#experimentList_schema
ChIP-peak Anno (Bioconductor)	Zhu L et al. (2010)^45^	https://bioconductor.org/packages/release/bioc/html/ChIPpeakAnno.html
Galaxy	Galaxy Community Hub	https://usegalaxy.org
Cis-regulatory element annotation system (CEAS; ChIPseeker, Bioconductor)	Xuwo Ji et al. (2006) Wang Q et al. (2015)	http://www.bioconductor.org/packages/release/bioc/html/ChIPseeker.html
Cellranger	10X Genomics	https://support.10xgenomics.com/single-cell-gene-expression/software/pipelines/latest/what-is-cell-ranger
Loupe Browser	10X Genomics	https://support.10xgenomics.com/single-cell-gene-expression/software/visualization/latest/what-is-loupe-cell-browser
Seurat	Hao Y et al. (2021)^46^	https://satijalab.org/seurat/
R	R Core Team (2021)	https://www.R-project.org/
Cell cycle score	Tirosh I et al. (2016)	https://satijalab.org/seurat/articles/cell_cycle_vignette.html
Prism 8.0	GraphPad	https://www.graphpad.com/scientific-software/prism/

**Other**		

35 mm glass bottom dish	Matsunami-grass	D11130H
Thermal Cycler Dice Real Time System II	Takara	TP900/TP960
Bioruptor	Cosmo Bio	UCD-300
Tape Station 2200	Agilent	G2964AA
NextSeq 500	Illumina	SY-415-1001
Chromium controller	10X Genomics	1000204
HiSeq X Ten	Illumina	SY-412-1001

### Resource availability

#### Lead contact

Further information and requests for resources and reagents should be directed to and will be fulfilled by the lead contact, Takashi Minami (t-minami@kumamoto-u.ac.jp).

#### Materials availability

All reagents generated in this study are available from the lead contact without restriction.

#### Data and code availability

• All next generation sequencing data and microarray data are available via NCBI Gene Expression Omnibus (GEO) database (GEO: GSE220509).
• This paper does not report original code.
• Any additional information required to reanalyze the data reported in this paper is available from the lead contact upon request.

### Experimental model and study participant details

#### Cell lines and cell culture

HUVECs were cultured in an EGM-2 MV bullet kit supplemented with 5% fetal bovine serum (FBS). HUVECs were passaged every 2 to 3 days and cells from passages 4−10 were used. Cos-7 and SH-SY5Y were cultured in Dulbecco's modified Eagle's medium supplemented with 10% FBS. All cells were cultured at 37°C at 5% CO_2_ in a humidified incubator. In treating HUVECs with hVEGF or hTNF-α, sub-confluent cells were washed once with Hank’s buffered saline (HBSS), cultured with EBM-2 supplemented with 0.5% FBS for 10 h, and treated with 20 ng/mL VEGF or 10 ng/mL TNF-α for the indicated time points. Chemical inhibitors were added 1 h before VEGF treatment as follows: 1 μM LB100, 50 μM LY294002, 30 μM SP600125, and 3 μM XMU-MP-1.

#### Transfection of siRNA

1 x 10^5^ HUVECs were plated in a 6-well plate and transfected with 20 nM siRNA using 2 μL/well Lipofectamine RNAi MAX. HUVECs were harvested 2 days after siRNA transfection.

#### Adenovirus transduction

HUVECs were infected with concentrated adenovirus (MOI: 100) and incubated for 48 h to transduce the vector harboring *FOXO1*. Human *FOXO1* was cloned and the mutated FOXO1 (constitutively nuclear-localized form) was created through mutagenesis by introducing mutations at T24A, S256A, and S319A. The mutated FOXO1 was subcloned into pIRES2-EGFP and then transferred to the Gateway™ pENTR™ 4 Dual Selection Vector system. To obtain adenovirus carrying *FOXO1* or mock control (EGFP alone), pAd/CMV/V5-DEST Gateway Vector Kit was used following the manufacturer's protocol. The virus titer was calculated using the Adeno-X Rapid Titer Kit.

### Method details

#### Real-time qPCR

Total RNA was extracted from HUVECs using ISOSPIN Cell & Tissue RNA Kit and was used for subsequent analysis. Real-time qPCR was performed using THUNDERBIRD® SYBR® qPCR Mix and Thermal Cycler Dice Real Time System II, following the manufacturer's instructions. RNA (500 ng) was reverse transcribed to cDNA using the PrimeScript RT Master Mix. The primers used are listed in Table S9.

#### Sprouting assay

siRNAs transfected HUVECs were seeded at 2,000 cells/well in a 96-well U-bottomed plate for 24 h and treated again with siRNAs for 12 h. Spheroids were collected, centrifuged at 2,000 × *g* for 5 s, and resuspended in 400 μL type I-A collagen gel. The spheroid-collagen solution was gently mixed and seeded on a 35 mm glass bottom dish. The plate was incubated for 30 min at 37°C, and then, the spheroids were cultured in 5 % FBS EGM-2 for 1 day for immunostaining or 2 days for calculation of the sprouting values.

#### Immunohistochemistry

Immunohistochemical analyses were performed using the formalin-fixed for 2 h and Protein blocker- 2 h preincubated HUVECs or mice retina. Specimens were incubated with 1st antibody at 4°C for one day and 1:100 dilution of Alexa Fluor dye-conjugated 2nd antibody at room temperature for 4 h as described previously.^26^^,^^47^ The 1^st^ antibodies used were: 1:100 dilution of FOXO1, FITC-conjugated CD31, CD31, and ESM-1.

#### RNA-seq

Total RNA was extracted from HUVECs using ISOSPIN Cell & Tissue RNA Kit. The libraries were prepared using TruSeq Stranded mRNA Library Prep Kit; 75 bp single-read sequencing was performed on Illumina NextSeq 500. The sequencing reads were aligned to the reference human genome (hg19) using STAR, and each transcript was counted using RSEM. To extract the differentially expressed genes (DEGs), an average cut-off values of log_2_ mean transcripts per million (TPM) between two samples >5.0 (18 h VEGF-treated HUVECs), >-2.5 (FOXO1 knockdown in HUVECs), and >-3.0 (adenoviral FOXO1 overexpression in HUVECs) were used. Genes with a value of log_2_-fold change >|1| (VEGF-treated HUVECs) and >|0.5| (FOXO1 knockdown and adenoviral FOXO1 overexpression in HUVECs) between two samples were considered to be significantly differentially expressed. The altered genes were clustered using Gene Cluster 3.0 and viewed in a heatmap using *Java Tree View* version 1.2.0. GSEA was performed using the software from Broad Institute.

#### ChIP

ChIP was performed using the Simple ChIP Enzymatic Chromatin IP Kit following the manufacturer's instructions. Briefly, HUVECs were cross-linked with 1% formaldehyde for 10 min at RT and neutralized with 0.2 M glycine. Nuclei were extracted using the lysis buffer; samples were digested using MNase for 20 min at 37°C and sonicated using the Bioruptor UCD-300 (5 cycles; 30 sec on /30 sec off). The extracted chromatin was immunoprecipitated with an anti-FOXO1 antibody at 1: 50 dilution. The prepared DNA was quantified using qPCR. The primers used are listed in Table S10.

#### ChIP-seq

ChIP sample libraries were prepared from NEB Next Ultra II DNA Library Prep Kit for Illumina, according to the manufacturer's instructions using 4 ng of ChIP sample or 1 mg of Input control. Library fragment sizes were evaluated on a Tape Station 2200, quantified using GenNext NGS Library Quantification kit, and sequenced using Illumina NextSeq 500. Sequencing reads were aligned to the reference hg19. MACS2 (Model-Based Analysis of ChIP-seq) was used to identify the enriched binding sites in the ChIP-seq data; a cut-off of *P* value <1 x 10^-4^ was used. Binding motifs were identified using the find Motifs Genome program of HOMER using default parameter input sequences comprising ±200 bp from the center of the peak.

Processed ChIP-seq data for FOXO1 (GSM1668935) in germinal center B cells,^34^ H3K4me3 (GSM2947421), H3K27ac (GSM2947429), and H3K27me3(GSM2947425) in HUVECs were downloaded from ChIP-Atlas.^43^ In addition, previously reported VEGF-treated H3K4me3, H3K9me3, H3K27me3, H3K27ac, and H2AK119Ub information (GSE159075)^26^ was used for further analysis. Computational processing in ChIP-Atlas is available at https://github.com/inutano/chip-atlas/wiki#experimentList_schema.^44^ Annotation of ChIP-seq peaks was performed using ChIP-peak Anno.^45^ FOXO1-ChIP enrichment in HUVEC around FOXO1-bound regions in B cells or vice versa was assessed using the computeMatrix tool and visualized using the plotHeatmap tool on the Galaxy platform (https://usegalaxy.org). FOXO1-ChIP enrichment in HUVEC around H3K4me3, H3K27Ac, or H3K27me3 peaks was visualized on the Galaxy platform (https://usegalaxy.org).

#### Single cell RNA-seq

##### Library preparation

0 or 18 h VEGF (20 ng/mL)-treated HUVECs were disassociated by Accumax. All libraries were prepared by following the 10x Genomics library preparation protocol and a total of 10,000 cells was loaded in this assay. The Chromium controller and Chromium NextGEM Single Cell 3' Kit v3.1 were used for library preparation. Sequencing was performed using HiSeq X Ten for 350 million read pairs/library with 28 bp first read and a 90 bp second read.

##### Data analysis- cell ranger

The raw fastq files were processed using the CellRanger 7.1.0 (10X Genomics) and mapped to a human reference dataset (refdata-gex-GRCh38-2020-A) (10X Genomics). We ran the cellranger count function with default parameters. Next, we aggregated two datasets (VEGF-0 h and VEGF-18 h) with the cellranger aggr function without depth normalization. The expression of various genes was analyzed with the Loupe Browser (10X Genomics) . Annotations of cell types identified with the Loupe Browser were exported to the downstream analysis.

##### Seurat

The Seurat v4 pipeline was used for library integration and differential expression analysis.^46^^,^^48^ The sctransform normalization, integration and dimension reduction of the dataset were executed following the Seurat Tutorial. Genes were filtered for expression in more than four cells. Then, cells were filtered for more than 2,500 genes and less than 9,000 genes, less than 70,000 UMIs, and more than one percent and less than ten percent mitochondrial reads. The top 30 principal components were used for the RunUMAP and FindNeighbors function. The FindClusters was performed using resolution 0.7. Cell types/states were annotated based on expression of several cluster-specific marker genes obtained with the FindAllMarkers function. The expression of cell-type specific genes was visualized with the FeaturePlot or VlnPlot commands from Seurat. Volcano plots were generated with EnhancedVolcano package. The UMAP coordinates calculated in Seurat were exported to analyze the expression of various genes with the Loupe Browser.

#### Plasmid construction and luciferase reporter assay

To generate the h*ESM-1 luciferase* (*luc*) plasmid, 1,230 bp *ESM1* promoter (from -1,161 to +69) and 1,011 bp enhancer (from -8,617 to -7,606) were cloned from the HUVEC genome; the resulting fragments were cloned into pGL4.10. For h*ANGPT2 luc* plasmid, 2,121 bp *ANGPT2* promoter (from -1,792 to +329) and 1,680 bp enhancer (from -37,268 to -35,588) were cloned into pGL4.10. The *luc* reporter assay was performed, as described previously.^47^ FuGENE HD or Lipofectamine 2000 transfection reagents were used for HUVEC or SH-SY5Y and Cos-7 cells, respectively, following the manufacturer's instruction.

#### Lentiviral CRISPR deletion of FOXO1 enhancer region

20 bp guide sequences (#1-5’; 5’-ACCTCTGACTGAGGCACGTT-3’, #1-3’; 5’-TGAAGTGTTAGGGCGCCTTT-3’, #2-5’; 5’-AGCTGGAGATGTGCCGCCAA-3’, #2-3’; 5’-CCGGCGGGCGGCTTCACGAC -3’) targeting the FOXO1-binding *ANGPT2* enhancer region were inserted in lentiviral dual-gRNAs packaging plasmid expressing with Cas9 protein (VB230331) and the high titer (>10^8^ TU/mL) lentiviruses were produced by VectorBuilder Inc. HUVEC were infected MOI = 100, and cultured over a week to fully express gRNAs and Cas9. The deleted genome was detected by using primers targeting *ANGPT2* enhancer region (No.1; FW 5’-CGGTCGTGAGGAATGTCGTT-3’, RV 5’-AGCGATTCTGAACGGTGCAT-3’, No.2; FW 5’-CACCAGAGACTCCGTGTACC-3’, RV 5’-CACTCACCCTTGATGGGAGC-3’) and PrimeSTAR Max.

#### Western blotting

Extraction and fractionation of nuclear and cytoplasmic proteins were performed as described previously.^49^ The antibodies used for the western blotting were as follows: FOXO1, Ser256 phosphorylated FOXO1, β-actin, lamin A, and β-tubulin. HRP-conjugated secondary antibodies used were anti-rabbit IgG and anti-mouse IgG.

### Quantification and statistical analysis

GraphPad Prism 8.0 was used for the analyses. The normality and variances of data were tested using appropriate tests such as the Kolmogorov-Smirnov test and *F* test; the values are expressed as mean ± S.D. All data passed the normality and equal variance tests. The *p*-value between two groups was calculated using the standard two-tailed unpaired *t*-test. Statistical significance between multiple samples was determined using 1- or 2-way ANOVA, to assess comparable variance followed by Tukey-Kramer or Dunnett's test. *P* < 0.05 was considered statistically significant.

## Supplemental information

Document S1. Figures S1–S10

Table S1. Dataset of up- and down-regulated genes by siFOXO1 treatment, related to Figure 2

Table S2. GSEA ranked list biological process and hallmark between siRNA to FOXO1 and control, related to Figure 2

Table S3. Dataset of up- and down-regulated genes by Ad-CA-FOXO1 treatment, related to Figure 2

Table S4. Dataset of up- and down-regulated genes by VEGF stimulation in 18 h, related to Figures 2 and 3

Table S5. Dataset of FOXO1-binding region by ChIP-seq analysis with FOXO1 antibody, related to Figure 4

Table S6. Dataset of FOXO1 binding genes in inversely correlated genes with siFOXO1 and Ad-FOXO1, related to Figure 4

Table S7. Gene list of GO analysis, related to Figures 4 and 5

Table S8. Gene list of FOXO1 binding in HUVEC and/or B-cell, related to Figure 6

Table S9. Gene list of GO analysis with FOXO1 binding genes in HUVEC and/or B-cell, related to Figure 6

Table S10. Primer sets for real-time qPCR and ChIP qPCR, related to key resources table
