# Supplementary material for: FOXO1 stimulates tip cell-enriched gene expression in endothelial cells
Source: iScience. 2024 Feb 16;27(3):109161. doi: 10.1016/j.isci.2024.109161 (PMC10914484; doi:10.1016/j.isci.2024.109161)
Supplement: Document S1. Figures S1–S10 [file mmc1.pdf]

iScience, Volume *27*

Supplemental information

FOXO1 stimulates tip cell-enriched gene expression in endothelial cells

Yuri Miyamura, Shunsuke Kamei, Misaki Matsuo, Masaya Yamazaki, Shingo Usuki, Keiichiro Yasunaga, Akiyoshi Uemura, Yorifumi Satou, Hiroto Ohguchi, and Takashi Minami

Figure S1

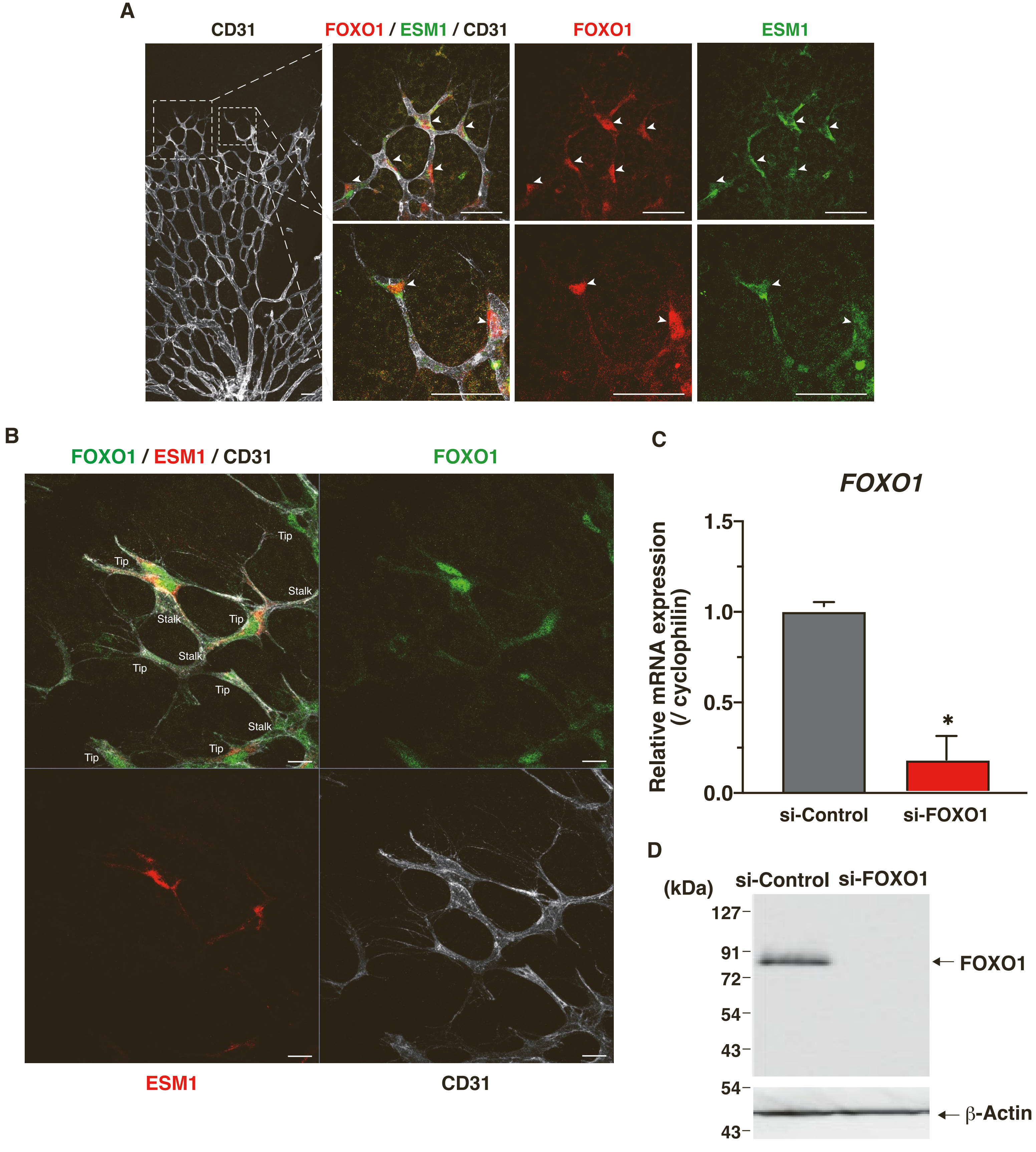

Figure S1 FOXO1 nuclear localized in tip cells, related to Figure 1. ***(A)*** left, Retinas from postnatal day-5 mice were dissected and immunostained with anti-CD31 antibody (*white*) to visualize the expression in the structures pertaining to blood vessel formation. Tip areas were enlarged to visualize the immunostaining with anti-FOXO1 (*red*) and anti-ESM1 (*green*). ESM1 was used as the representative tip marker. Arrowheads indicate FOXO1 co-localization with ESM1-positive cells. Scale bar, 50 μm. (***B***) Enlarged images of the dissected retina from an independent sample. Immunostaining was performed with antibodies against FOXO1 (*green*), ESM1 (*red*), and CD31 (*white*). The Upper left shows a merged image with tip vs. stalk indications. Bar: 50 μm. (***C***) Relative expression of FOXO1 in HUVECs treated with indicated siRNAs, quantified using qPCR. Data are represented as mean ± S.D., n=5. *P< 0.01 compared with si-Control. (***D***) Immunoblot of anti-FOXO1 antibody in HUVECs treated with indicated siRNAs. β-Actin indicates the loading control. The data was the representative for three independent experiments.

Figure S2

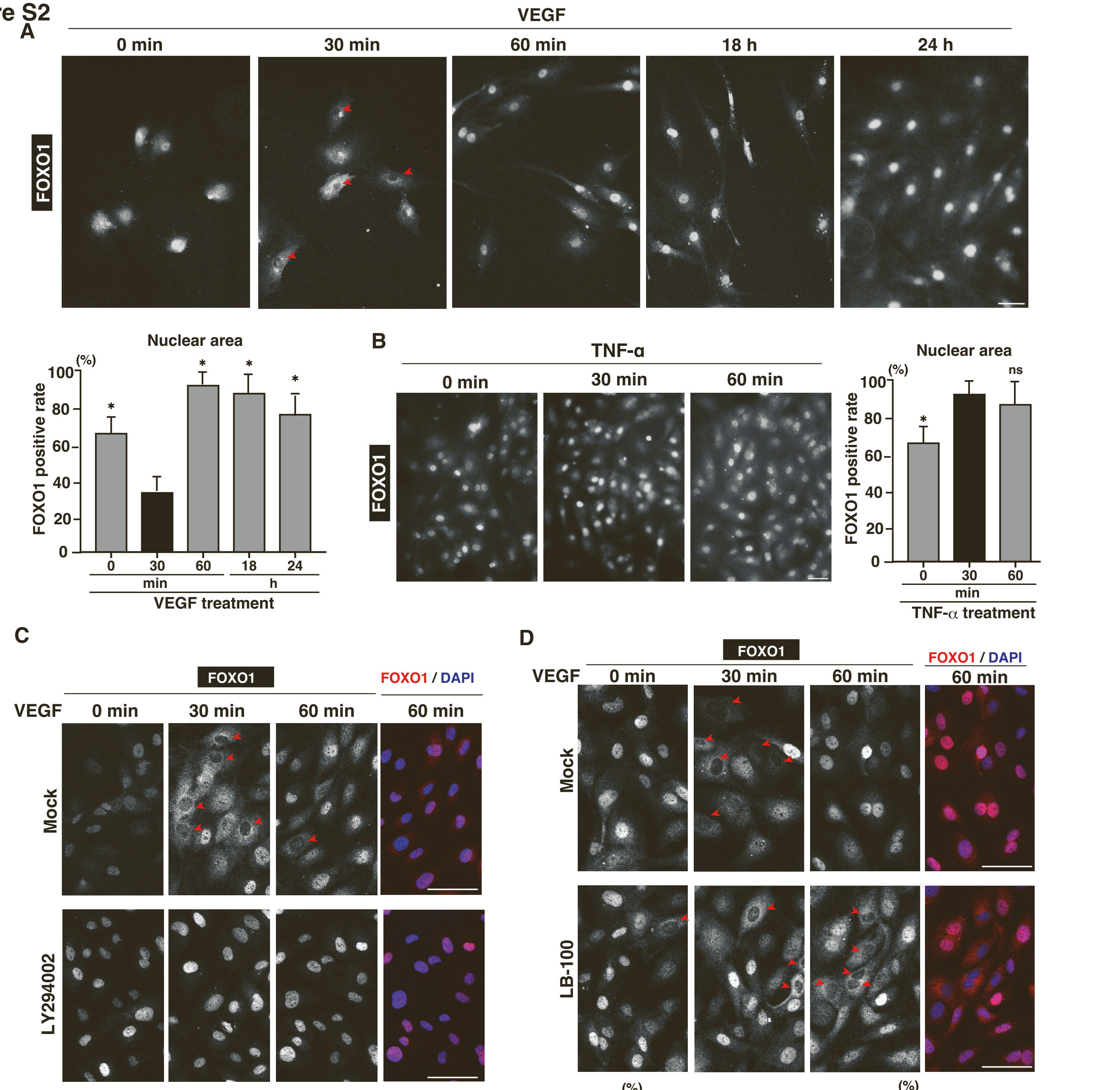

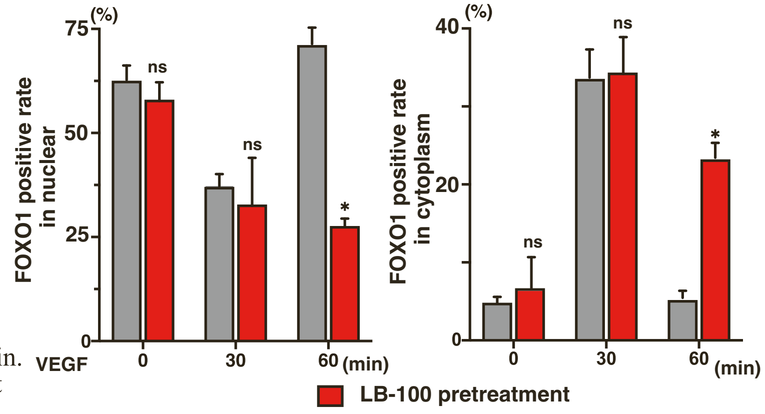

Figure S2 Dynamics of nuclear-cytoplasmic localization of FOXO1 in activated ECs, related to Figure 1. (*A*) Immunostaining of HUVECs treated with VEGF for 0, 30, 60 min, 18, and 24 h using anti-FOXO1 antibody. *Red* arrowheads indicate the cells that FOXO1 was extruded from the nuclear. Bar: 50 μm. The ratio of FOXO1 localized specifically to the nuclear regions in HUVECs was quantified using immunostaining. Data are shown as mean ± S.D. from more than 10 optical images from at least 8 independent experiments. *$P < 0.005$ compared with cells treated with VEGF for 30 min (lower graph). (***B***) Immunostaining with anti-FOXO1 antibody in HUVECs at 0, 30, and 60 min of TNF-α treatment. Bar: 50 μm. The ratio of nuclear to cytoplasmic localized FOXO1 in TNF-α treated HUVECs was quantified and shown in the bar graph. Data are shown as mean ± S.D., n=4.*$P<0.05$ compared with treatment of TNF-α for 0 or 60 min. (***C***) Immunostaining in HUVECs at indicated time points of VEGF treatment inthe presence or absence of the PI3K-AKT inhibitor, LY294002. Immunostaining with only the anti-FOXO1 antibody (*gray*) or merged with DAPI (shown on the *left*). Data are representative of four independent experiments. Arrowheads in *red* indicate the cells in which FOXO1 is completely localized in the cytosol. Bar: 50 μm. (***D***) *Upper*, immunostaining of HUVECs at indicated time points following VEGF treatment in the presence or absence of the PP2A inhibitor, LB-100. Immunostaining with anti-FOXO1 antibody (*gray*) is shown in the *right* column, and the merge with DAPI staining is shown in the *left* column. Data are representative of four independent experiments. Arrowheads in *red* indicate the cells in which FOXO1 was predominantly localized to the cytosol. Bar: 50 μm. *Lower*, the ratio of FOXO1 localized in the nucleus was quantified from the immunostaining images in the presence or absence of LB-100 pretreatment. Data are indicated as mean ± S.D. from more than 10 images from at least four independent experiments. *$P < 0.005$ compared with cells treated with VEGF for 60 min. ns, not significant.

Figure S3

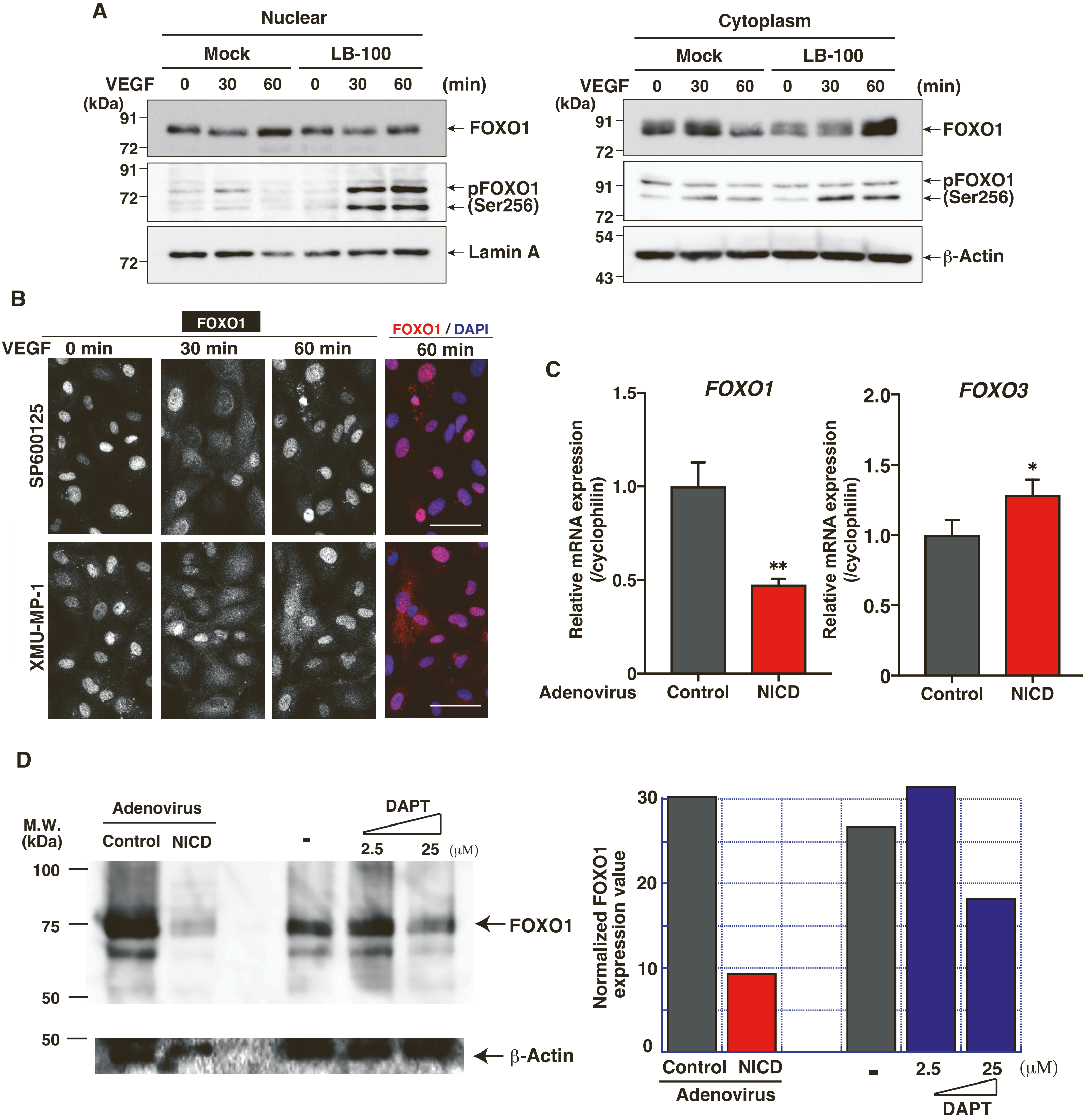

Figure S3 VEGF and Notch signal regulate the FOXO1, related to Figures 1 and 2. (***A***) The total FOXO1 protein (upper) and Ser256-phosphorylated FOXO1 protein (*middle*) assessed using western blotting of HUVEC samples treated for 0, 30, and 60 min with VEGF. The cells were lysed and separated into nuclear (left) and cytoplasmic (right) fractions. Lamin A and β-Actin were used as the nuclear and cytoplasmic internal control, respectively. The data are representative of three independent experiments. (***B***) Immunostaining in HUVECs at indicated time points of VEGF treatment in the presence or absence of the JNK inhibitor SP600125, or the Mst1/2-kinase inhibitor XMU-MP-1. Immunostaining with only the anti-FOXO1 antibody (*gray*) or merged with DAPI (shown on the *left*). Data are representative of three independent experiments. Bar: 50 μm. (***C***) HUVECs were treated with either Ad-Control or Ad-NICD for 24 h, and the harvested RNA was subjected to qPCR with FOXO1 and FOXO3 specific primers. The results show the mean ± S.D. relative to cyclophilin A. $^{**}P<0.01$, and $^{*}P<0.05$ compared with Ad-Control. (***D***) HUVECs were treated with either Ad-Control or Ad-NICD, or indicated doses of DAPT for 2 days. Immunoblots were performed with anti-FOXO1 antibody. β-Actin were used as the internal control The specific band intensities were quantified and shown the average in *left* bar graph (n=2).

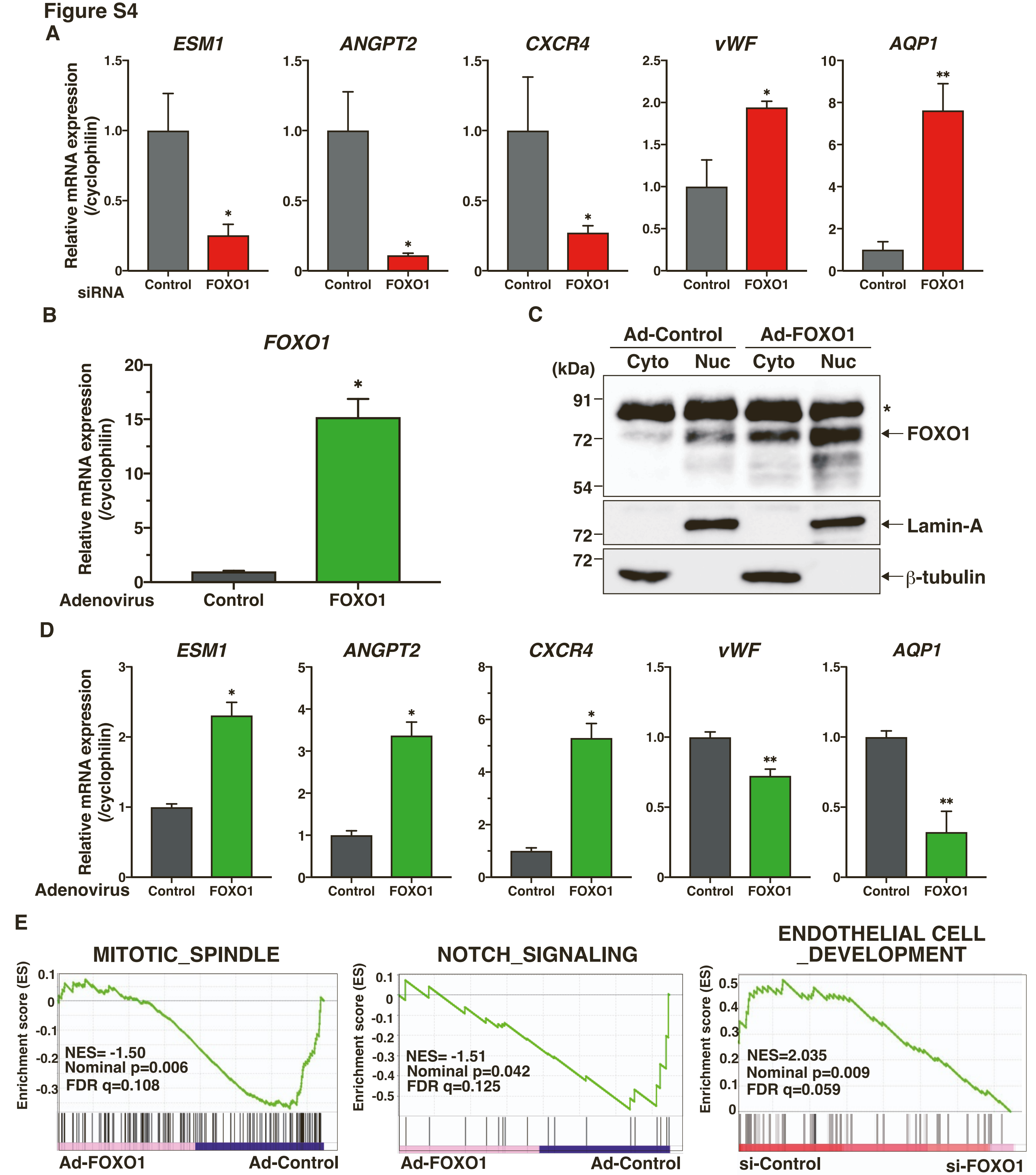

Figure S4 siRNA to FOXO1-or Ad-FOXO1-mediated gene expression changes in the endothelium, related to Figure 2. (***A***) Relative expression of indicated genes in HUVECs treated with siRNAs, quantified using qPCR. Data are represented as mean ± S.D., n=3. **P*< 0.05 and ***P*<0.005 compared with si-Control. (***B***) Relative expression of FOXO1 in HUVECs treated with Ad-Control or constitutively nuclear-localized FOXO1, quantified using qPCR. Data are represented as mean ± S.D., n=4. **P*< 0.005 compared with Ad-Control. (***C***) Western blotting for FOXO1 in HUVECs treated with adenoviruses. The extracted proteins were divided into the cytosolic (Cyto) and nuclear (Nuc) fractions. Lamin A and β-tubulin were used as the internal loading control for the nuclear and cytosolic fractions, respectively.*; nonspecific band. Data are representative of three independent experiments. (***D***) Relative expression of indicated genes in HUVECs treated with adenoviruses, quantified using qPCR. Data are represented as mean ± S.D., n=3. * *P*< 0.01 and ** *P*<0.05 compared with Ad-Control. (***E***) GSEA of Ad-FOXO1 or si-FOXO1 in HUVECs. Gene signatures for cell proliferation; mitotic spindle, and Notch signaling are shown with Ad-FOXO1 vs the control. Gene signature unique to EC development is shown with siRNA to FOXO1 vs. the control.

Figure S5

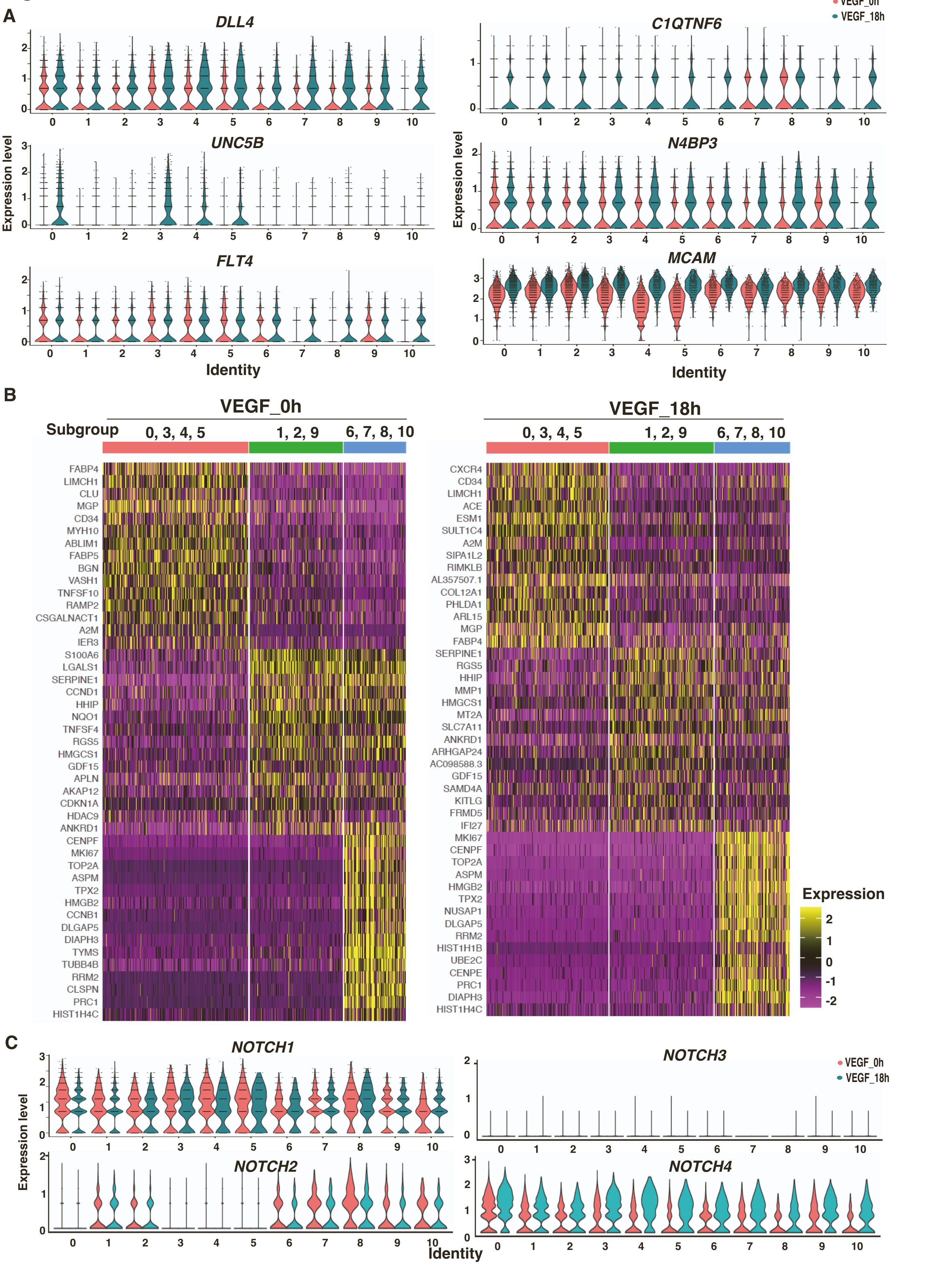

Figure S5 Single cell RNA-seq mediated subdivision of primary cultured endothelium with tip-or stalk-enriched gene set, related to Figure 3. (***A***) Violin plots of expression value for tip enriched genes in 10 differential subpopulations, related to Fig. 3E (*red*, 0h; *green* 18h of VEGF treatment). (***B***) Heat map of pearson correlation was shown with top differentially expressed genes (DEGs,top 4 to 5 genes) among the 10 subpopulations (Fig. 3A) in 0h and 18h VEGF-treated HUVECs. Log-fold expression values are shown with higher (*yellow*) and lower (*purple*) relative to median (*black*). (C) Violin plots of expression value for NOTCH genes in differential EC subpopulations (*red*, 0h; *green* 18h of VEGF treatment).

Figure S6

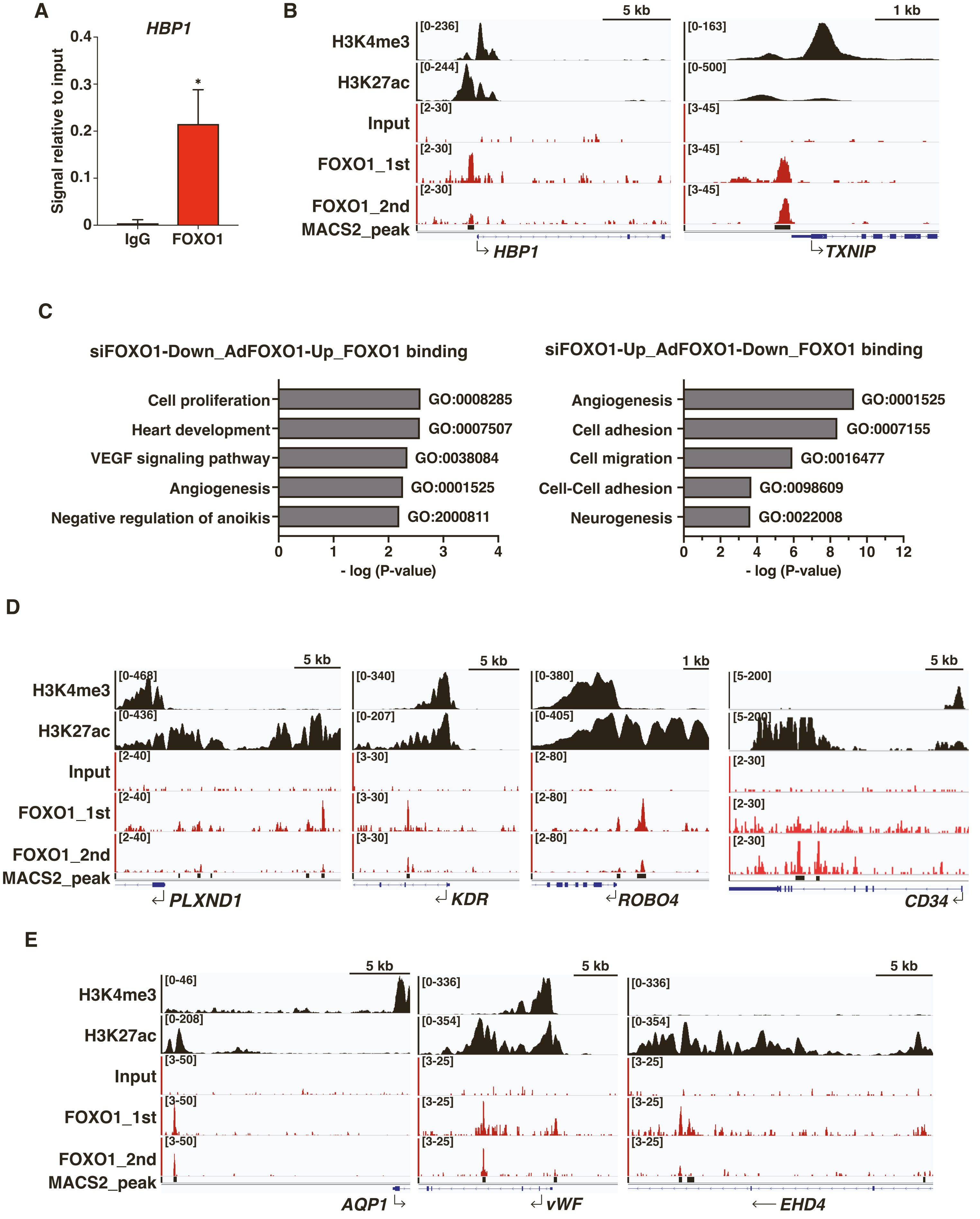

Figure S6 Global endogenous FOXO1-ChIP-seq analysis from VEGF-treated endothelium, related to Figures 4 and 5. (***A***) ChIP quality validation using anti-FOXO1. HBP1 is the representative FOXO1-bound gene. ChIP-qPCR of HBP1 loci in VEGF-treated HUVECs normalized to the input. Data are shown as mean ± S.D., n=3. **P*<0.05 compared with the enrichment value from control IgG. (***B***) Integrated genome view of HBP1 and TXNIP, the known FOXO1 bound target genes. (***C***) DAVID GO analysis of FOXO1 bound genes with either si-FOXO1 reduced and Ad-FOXO1 induced (*left*) or si-FOXO1 induced and Ad-FOXO1 reduced (*right*) conditions. (***D***) Integrated genome view of tip-marked EC-specific genes. (***E***) Integrated genome view of stalk-marked genes.

Figure S7

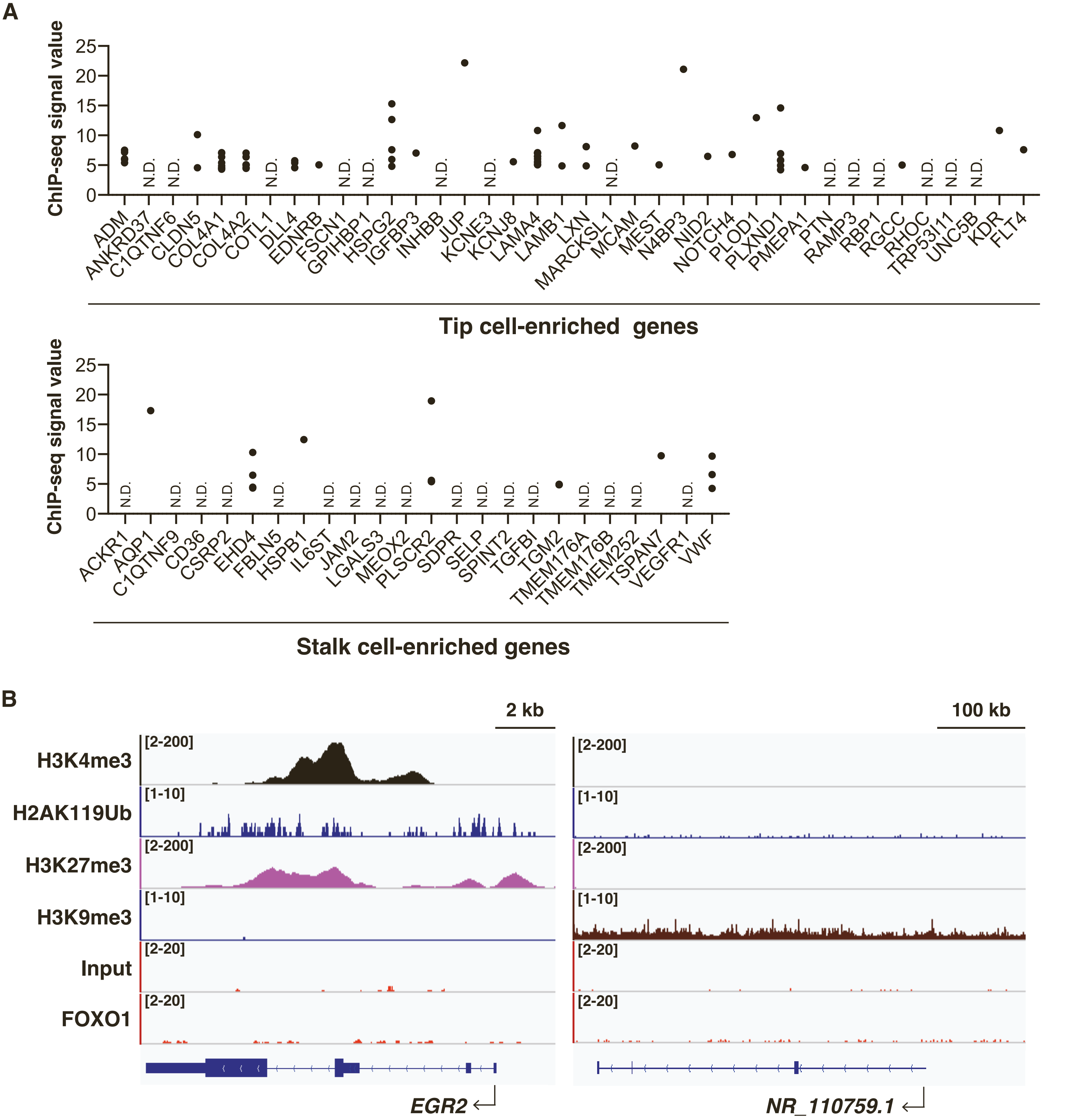

Figure S7 FOXO1 predominantly binds tip-marked genes with pre-active chromatin microenvironment, related to Figure 4. (***A***) FOXO1-ChIP-seq enrichment values are quantified with each tip vs. stalk mark categorized gene loci derived from (ref. 38). N.D.: not detected. (***B***) Integrated genome view of the representative EC-specific bivalent gene, EGR2, and the H3K9me3 interacting gene adjacent to the centromere, NR_110759.1.

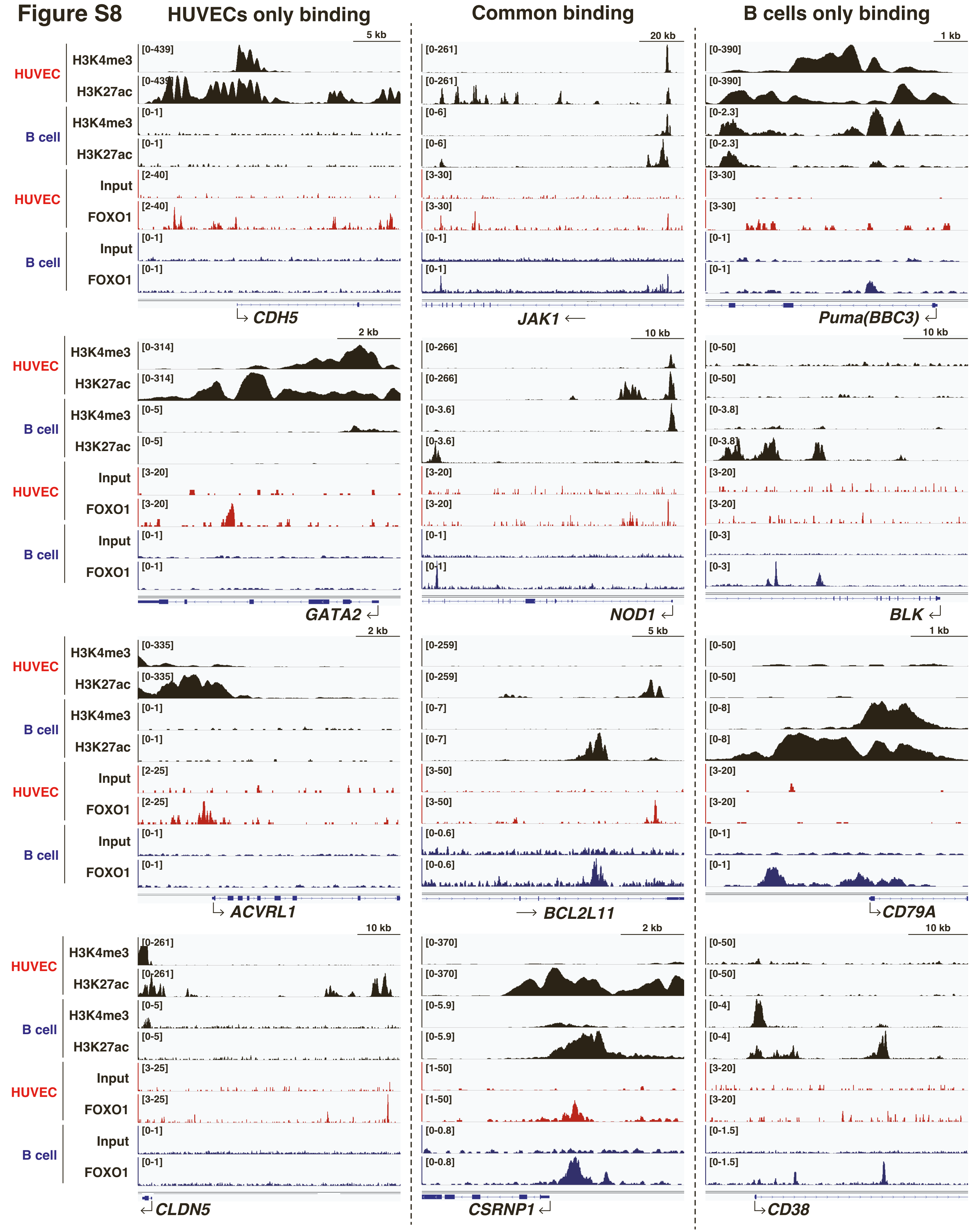

Figure S8 Cell-type specific FOXO1 binding patterns are observed with FOXO1 expressed in each cell, related to Figure 6. Integrated genome view of the representative gene loci specific to HUVECs or B cells and those common in both cells with FOXO1 enrichments.

Figure S9

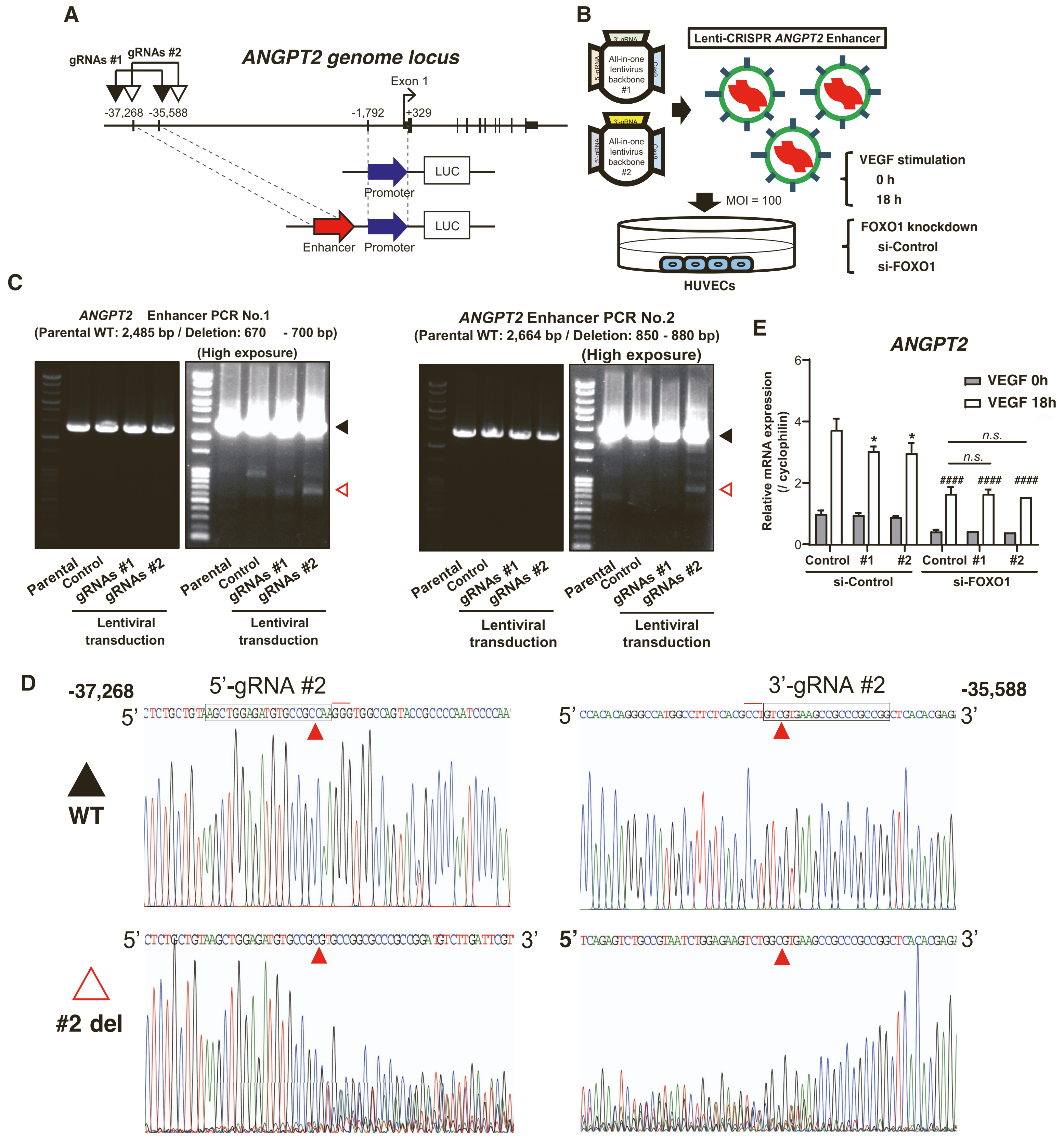

Figure S9 Lentiviral CRISPR deletion of FOXO1-binding ANGPT2 enhancer region modestly decreased ANGPT2 mRNA expression in VEGF-treated endothelium, related to Figure 7. (*A*) Schematic representation of the targeting site of CRISPR guide RNAs to FOXO1-bound H3K27ac-enriched enhancer on ANGPT2 locus. (*B*) Experimental design of lentivirus-mediated CRISPR guide RNAs and Caspase 9 transduction into the HUVECs. (*C*) PCR validation of site deletion by CRISPR gRNAs. 2 independent PCR primer sets were used. The black arrowhead indicates intact (WT) and the red arrowhead with a dotted line indicates expected PCR products of ANGPT2 enhancer-deleted genome. (*D*) Sanger sequencing of around #2 guide RNAs-targeting site on ANGPT2 enhancer region. The PCR products of black (WT) and red (enhancer deletion) arrowheads of #2 gRNAs-transduced HUVEC cells in (*C*) were shown. *Red* arrowheads in the peak map indicate the cleavage site of CRISPR-Cas9. (*E*) Relative expression of ANGPT2 quantified using qPCR. siRNAs to control or FOXO1 treated HUVECs were cultured in the presence or absence of VEGF. *, $p<0.05$, vs. lentiviral control; ####, $p<0.0001$, vs. si-control with 18 h VEGF treatment; n.s., not significant.

Figure S10

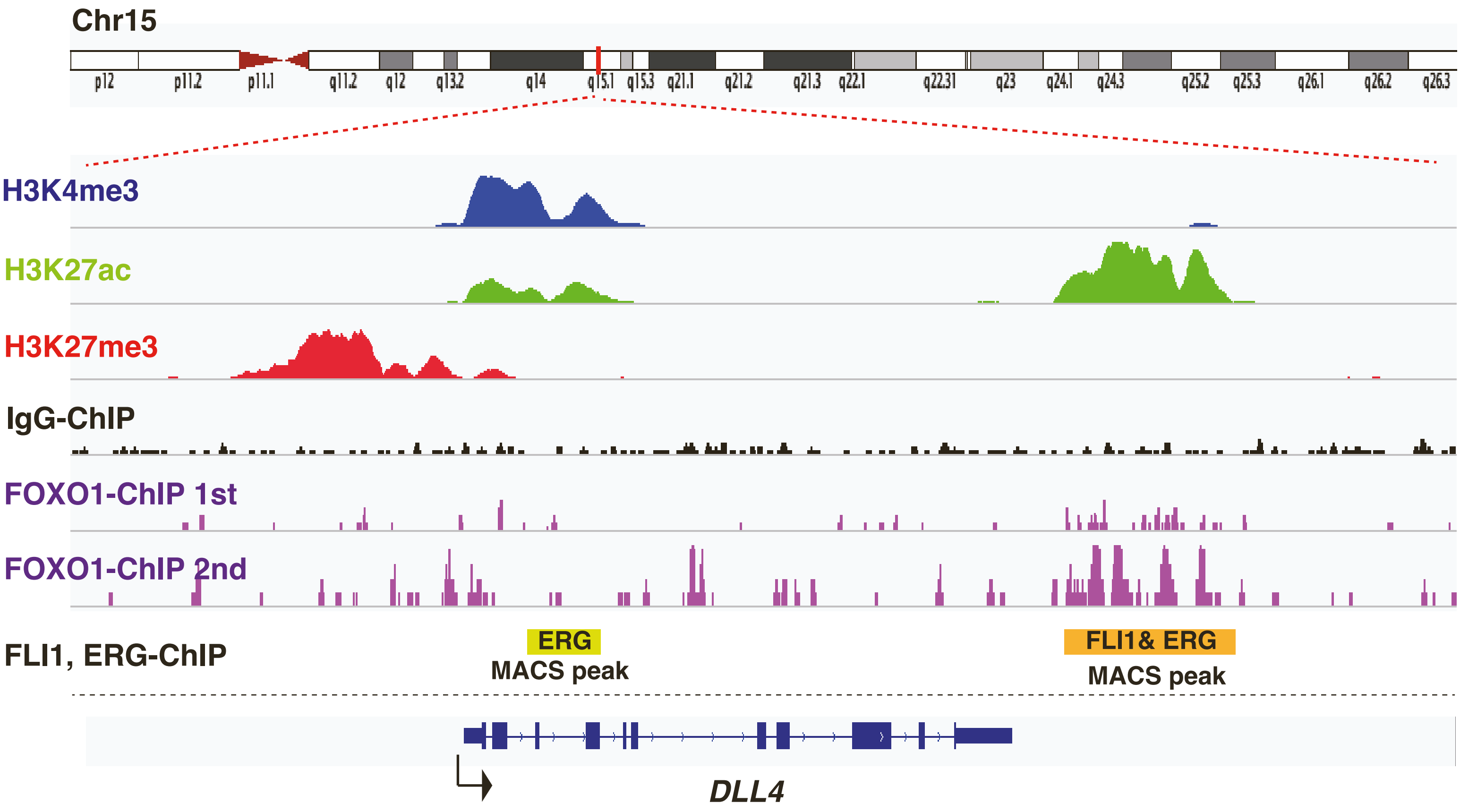

Figure S10 Unique histone map of hDLL4 on the Chr.15, related to Figure 5. Active promoter mark; H3K4me3 (*blue*), enhancer mark; H3K27ac (*green*), and the differenced region of silencer mark; H3K27me3 (*red*) were shown in hDLL4 locus. Enriched signals of two-independent endogenous FOXO1-ChIP seqs were shown in purple. MACS calculated-positive signal for ERG binding was shown in the yellow, and the ERG and FLI1 common binding was *orange* rectangle, respectively.
